# A new species of branchial fish parasitic deep-sea isopod, *Brucethoa* Aneesh, Hadfield, Smit & Kumar, 2020 (Isopoda: Cymothoidae) from the Indian Ocean, with the transfer of two *Elthusa* Schioedte & Meinert, 1884 species

**DOI:** 10.1007/s11230-024-10149-0

**Published:** 2024-03-13

**Authors:** Panakkool Thamban Aneesh, Ameri Kottarathil Helna, Appukuttannair Biju Kumar

**Affiliations:** 1 Blue Innovation Division, Seto Inland Sea Carbon Neutral Research Center, Graduate School of Integrated Sciences for Life, Hiroshima University, 5–8–1 Minato-machi, Trivandrum, Hiroshima 725–0024 Japan; 2 Travancore Nature History Society (TNHS), MBRRA, Mathrubhumi Road, Vanchiyoor, Trivandrum, Kerala 695035 India; 3 Regional Forensic Science Laboratory, Kannur, Kerala 670002 India; 4 Department of Aquatic Biology and Fisheries, University of Kerala, Karyavattom, Thiruvananthapuram, Kerala 695 581 India

## Abstract

*Brucethoa* *isro* **n. sp.**, a new species of deep-sea cymothoid is described and illustrated from the host fish Spinyjaw greeneye, *Chlorophthalmus corniger* Alcock, 1894, at depths of 265 to 458 metres from the southwest coast of India. *Brucethoa* *isro* **n. sp.** is recovered from the base of the gill cavity, facing the head towards the anterior, and the dorsal body closely adpressed against the gill, while the ventral brood presses against the inner wall of the operculum. *Brucethoa isro* **n. sp.**, the second species of the genus, is characterized by: head weakly immersed in pereonite 1, very elongated body (3.15 times as long as wide); body dorsum not vaulted, almost flat; all coxae short, 0.5 times as the length of corresponding pereonites; sternite 7 with prominent posterior lobes. All adult life stages of the new species are described [including females (ovigerous and non-ovigerous), males, transitional, and juvenile. The species is currently known from the southwest coast of India and is the type locality. Additionally, this research provides valuable ecological insights into *Brucethoa isro* **n. sp.** and its habitat. As part of the taxonomic contributions, two species, *Brucethoa alvaradoensis* (Rocha-Ramírez, Chávez-López & Bruce, 2005) **comb. n.** and *Brucethoa epinepheli* (Trilles & Justine, 2010) **comb. n.,** are transferred from the *Elthusa* genus to the *Brucethoa* genus.

## Introduction

Fish parasitic isopod family Cymothoidae Leach, 1814 globally includes 365 species under 41 valid genera (Aneesh et al., 2023b). Among them, eleven genera are monotypic. More than 70 % of the species (282 out of 365) are from the following eight genera: *Anilocra* Leach, 1818 (61 species); *Ceratothoa* Dana, 1852(26 species); *Cymothoa* Fabricius, 1793 (42 species); *Elthusa* Schioedte & Meinert, 1884 (41 species); *Ichthyoxenos* Herklots, 1870 (19 species); *Mothocya* A. Costa *in* Hope, 1851 (30 species); *Nerocila* Leach, 1818 (44 species); *Renocila* Miers, 1880 (20 species) (Aneesh et al., 2016; Kawanishi et al., 2023). Nineteen genera with less than five species, at least 15 genera are known to infest the branchial cavity, including the recently described *Brucethoa* Aneesh, Hadfield, Smit, & Kumar, 2020 and *Glyptothoa* Helna, Aneesh, Kumar & Ohtsuka, 2023 (Aneesh et al., 2020b, 2023a; Helna et al., 2023).

Indian cymothoid fauna is well-documented, with 62 valid species under 22 genera (Ravichandran et al., 2019; Aneesh et al., 2020a, 2021a, 2022a, 2023a; Helna et al. 2023). Among them, at least 18 species are originally described from Indian waters, which indicates the scope of exploration and description of more new taxa, especially from non-commercial and deep-water hosts. Parasitic cymothoids infesting commercial fish from Indian waters are well documented (Ravichandran et al., 2019; Aneesh et al., 2020a, b, 2021a, b, 2022a, b, 2023b; Nashad et al., 2022). On the other hand, cymothoid infestation of non-commercial deep-sea- trash fishes is not well documented.

The marine fish Spinyjaw greeneye, *Chlorophthalmus corniger* Alcock, collected at depths of 265 to 458 metres from the southwest coast of India, and the occurrence of a branchial parasitic isopod, specifically attached to the base of the gill cavity, facing the head towards anterior, with some unique morphology, always creates curiosity among taxonomists. While identifying the present cymothoid specimen, it was clear that it belonged to the recently described genus *Brucethoa*, by the following characteristics: cephalon with a rostral point; bilobed brood plate 1; sternite 7 with prominent posterior lobes; larger pleopods. Accordingly, we described a new species of *Brucethoa* in the present study based on the following life stages, female, male, transitional, and juvenile. The ecological remarks of the new species are also provided. The two *Elthusa* species transferred to *Brucethoa* are: *B. alvaradoensis* (Rocha-Ramírez, Chávez-López & Bruce, 2005) **comb. n.**, and *B. epinepheli* (Trilles & Justine, 2010) **comb. n.**

## Materials and methods

Fresh specimens of unidentified cymothoid samples were collected from the branchial cavity of the deepsea fish Spinyjaw greeneye, *Chlorophthalmus corniger* Alcock (Aulopiformes: Chlorophthalmidae), from Neendakara (08^0^30.0′N 76^0^53.30′E), Kollam district, Kerala state, southwest coast of India. The collected cymothoids were processed following the techniques described by Aneesh et al., (2019, 2021c). One ovigerous female was designated as the holotype, and one paratype and a few non-type specimens were minimally dissected to conserve the specimens (the dissected appendages were kept in separate vials along with the said specimen). Methods for dissection, mounting, and drawings of appendages were according to the techniques described in Aneesh et al., (2019). The specimens were microphotographed using a multi-focusing dissection microscope Leica-M205A and image capturing software (Leica Application Suit). Drawings were digitally inked using Adobe Illustrator and a WACOM CintiQ DTK-1300 drawing pad. Distribution of *Brucethoa bharata* Aneesh, Hadfield, Smit & Kumar, 2020 and *Brucethoa isro* **n. sp.** is provided in Fig. 22. Sources for the fish taxonomy and host nomenclature were Fish Base (Froese & Pauly, 2023) and Catalogue of Fishes (Fricke et al., 2023). The type specimens are deposited in the Western Ghat Field Research Centre of the Zoological Survey of India, Kozhikode (ZSI/WGRC) and other collections are deposited in PTA and AKH’s personal collection, extended in India (CAH).

*Abbreviations:* **RS **robust seta/e; **BL **body length; **W **width.

**Taxonomy**

**Suborder Cymothoida Wägele, 1989**

**Superfamily Cymothooidea Leach, 1814**

**Family Cymothoidae Leach, 1814**

**Genus** ***Brucethoa*** **Aneesh, Hadfield, Smit & Kumar, 2020**

*Brucethoa* Aneesh, Hadfield, Smit & Kumar, 2020: 566.— Aneesh, Helna & Kumar, 2021: 343.

***Type species:*** *Brucethoa bharata* Aneesh, Hadfield, Smit & Kumar, 2020.

**Remarks: **Aneesh et al., (2020b) recently described the deep sea cymothoid genera *Brucethoa* as a monotypic genus for *B. bharata*. The following combinations of characters characterise the genus: cephalon weakly immersed in pereonite 1, cephalon anterior margin with acute ventrally directed rostral point, pereonites 6 and 7 with posterolateral margin expanded; pleon with gaps between the pleonites; both antennae slender, with antennula shorter than antenna, and large pleopod rami that extend laterally beyond the pleotelson margins; ovigerous females have proximally thickened bilobed oostegite 1, and the marsupium is posteriorly partly enclosed by two sub-rectangular fleshy lobes. A detailed generic diagnosis and comparison of *Brucethoa* with other branchial cymothoid genera were provided by Aneesh et al., (2020b).

**Species included:** *Brucethoa alvaradoensis* (Rocha-Ramírez, Chávez-López & Bruce, 2005) **comb. n.;** *Brucethoa bharata* Aneesh, Hadfield, Smit & Kumar, 2020; *Brucethoa isro* **n. sp.**; *Brucethoa epinepheli* (Trilles & Justine, 2010) **comb n.**

*Brucethoa isro*** n. sp.**

(Figs. 1, 2, 3, 4, 5, 6, 7, 8, 9, 10, 11, 12, 13, 14, 15, 16, 17, 18, 19, 20)

**Fig. 1:**
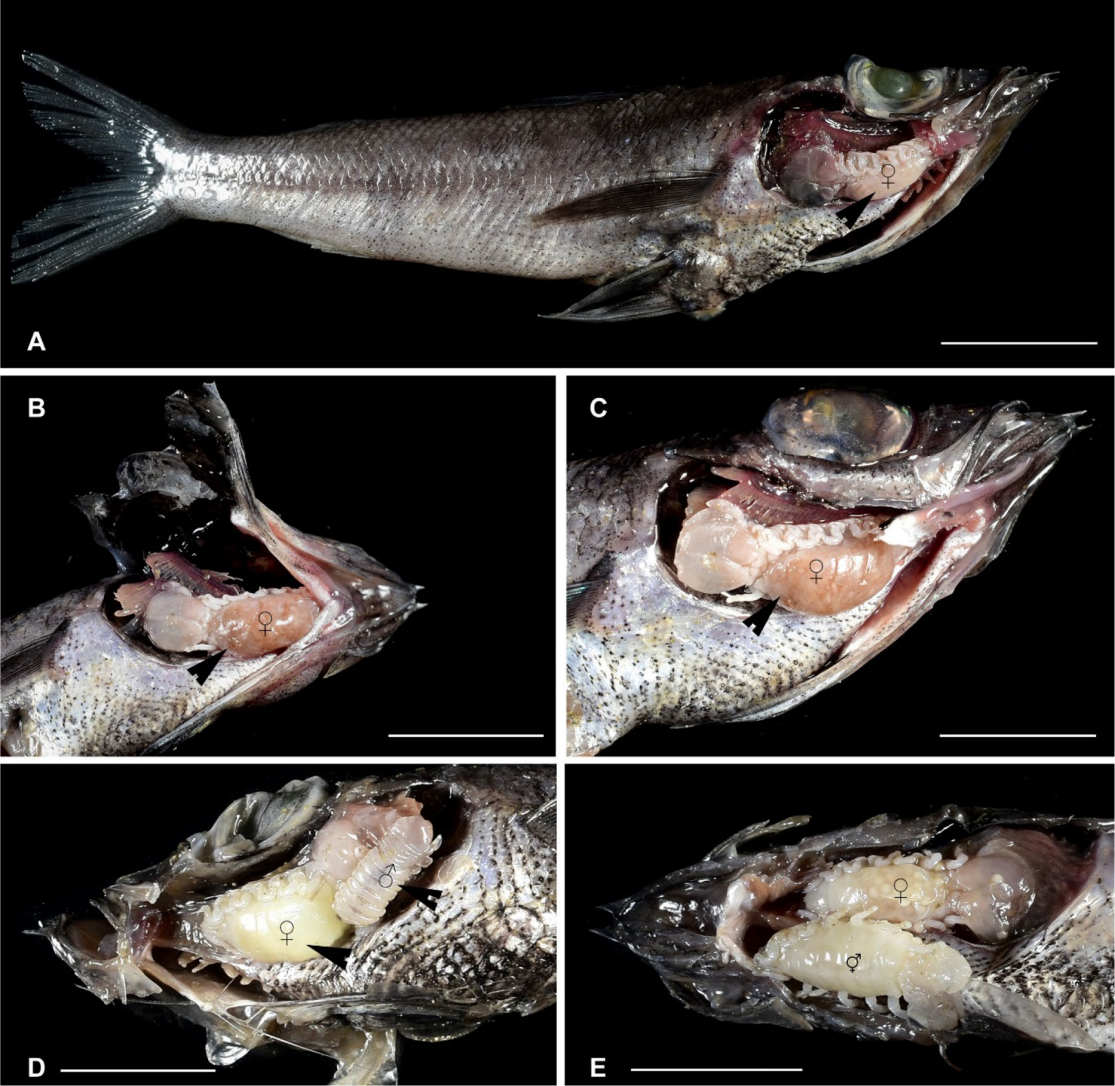
**A**–**E,** *Brucethoa isro* **n. sp.,** ovigerous female on the branchial cavity of the host fish Spinyjaw greeneye, ***Chlorophthalmus corniger*** Alcock. *Scale, A: 20 mm; B–E: 10 mm.*

**Fig. 2:**
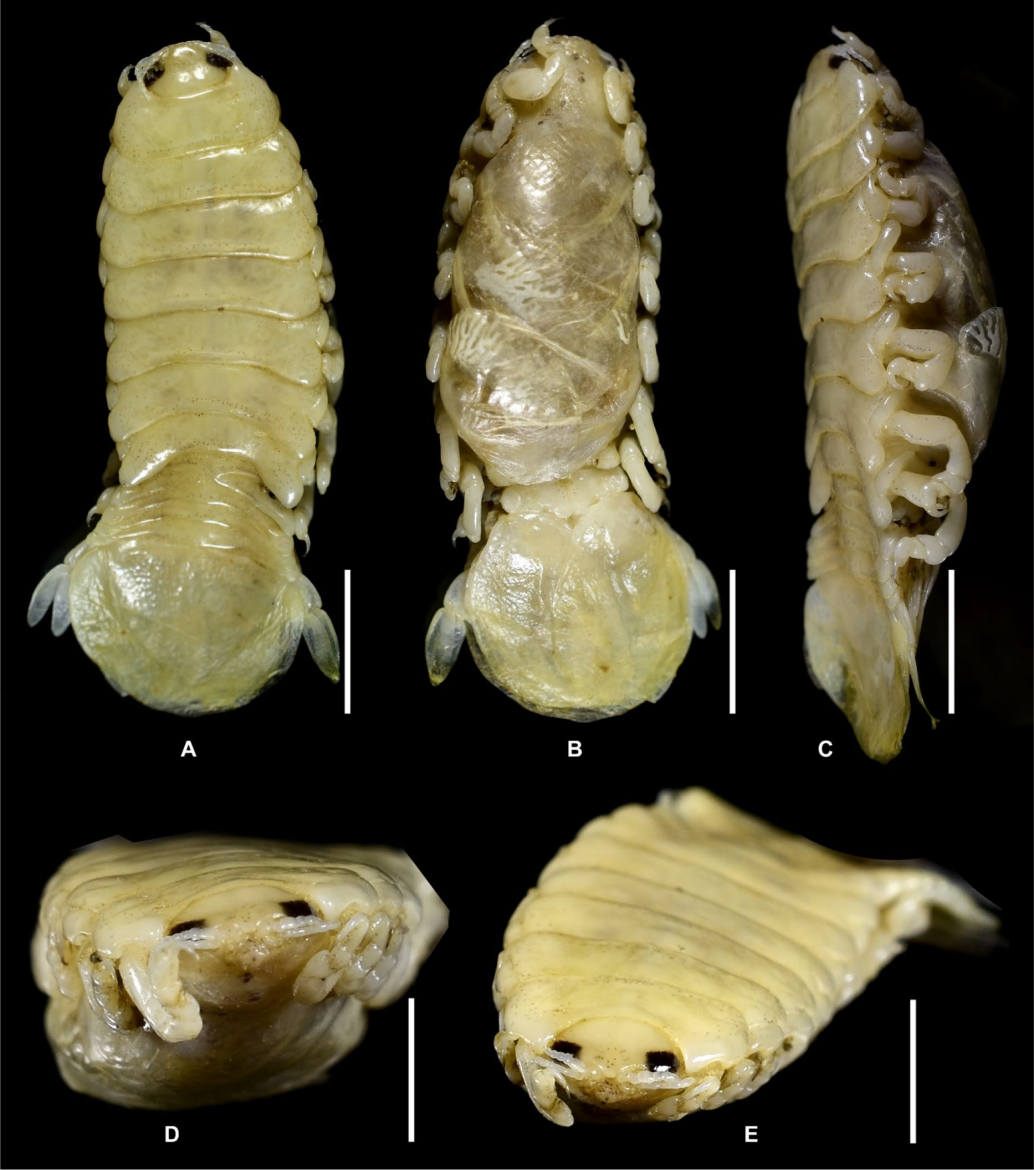
*Brucethoa isro* **n. sp.,** ovigerous female holotype (Reg. No. ZSI/WGRC/IR. INV/26312). A, dorsal view; B, ventral view; C, lateral view; D–E, dorso-frontal view. *Scale, A – E: 5 mm.*

**Fig. 3:**
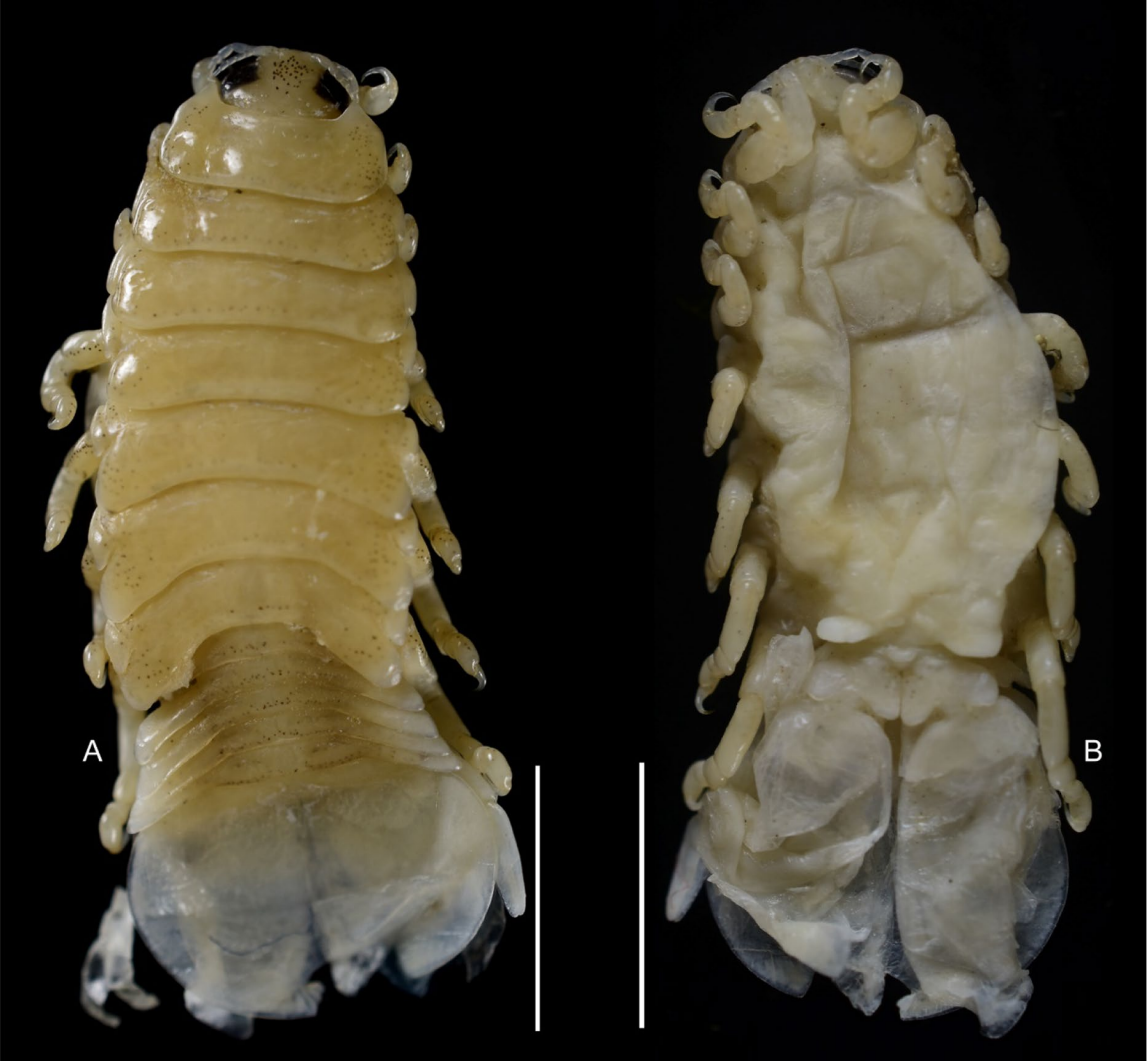
***Brucethoa isro*** **n. sp.,** non-ovigerous female paratype (Reg. No. ZSI/WGRC/IR. INV/26315) A, dorsal view; B, ventral view. *Scale, A – B: 5 mm.*

**Fig. 4:**
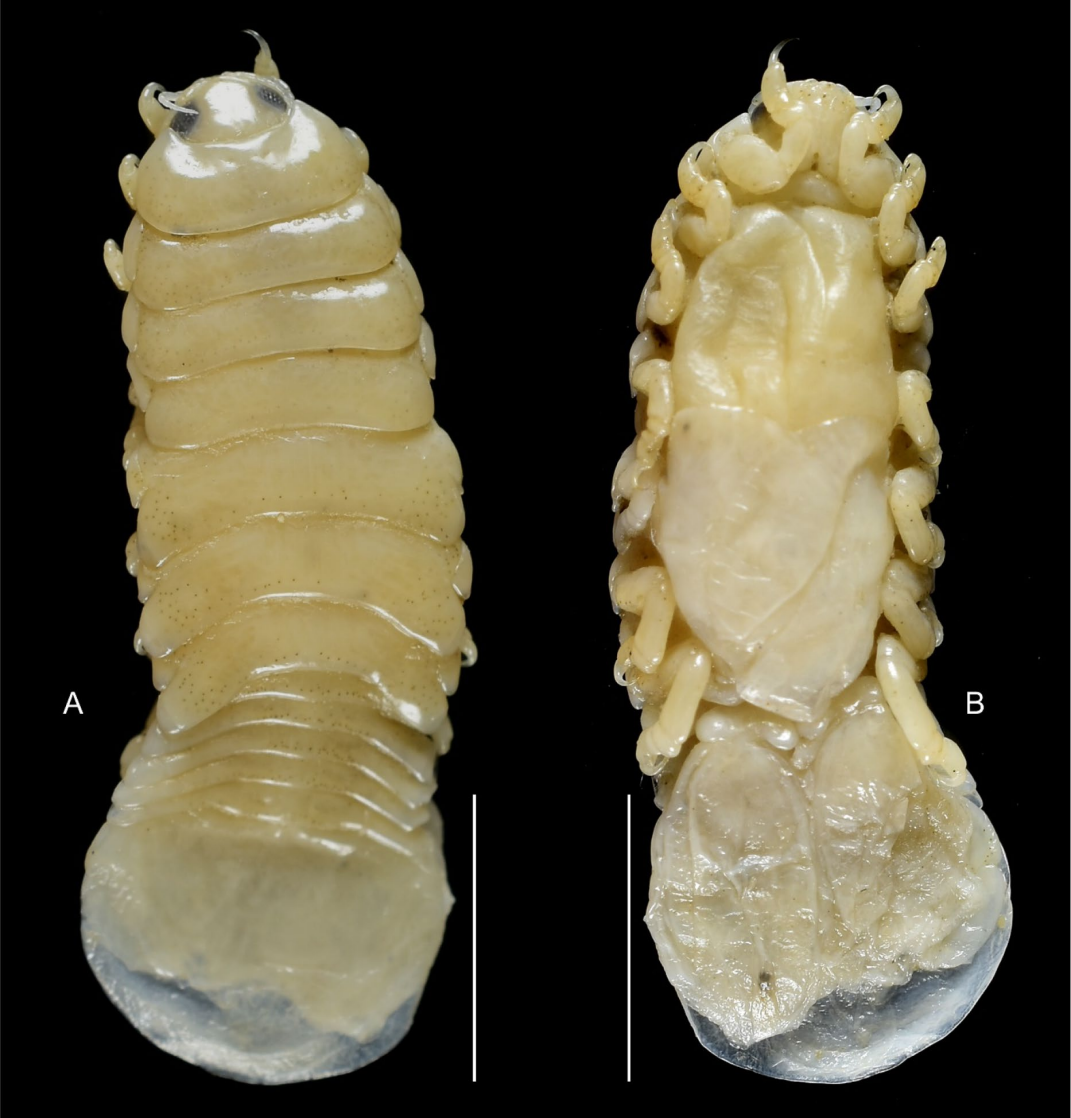
*Brucethoa isro* **n. sp.,** non-ovigerous female on moulting (oostegition moult) (Reg. No. CAH/INV/ISO 0302). A, dorsal view; B, ventral view. *Scale, A – B: 5 mm.*

**Fig. 5:**
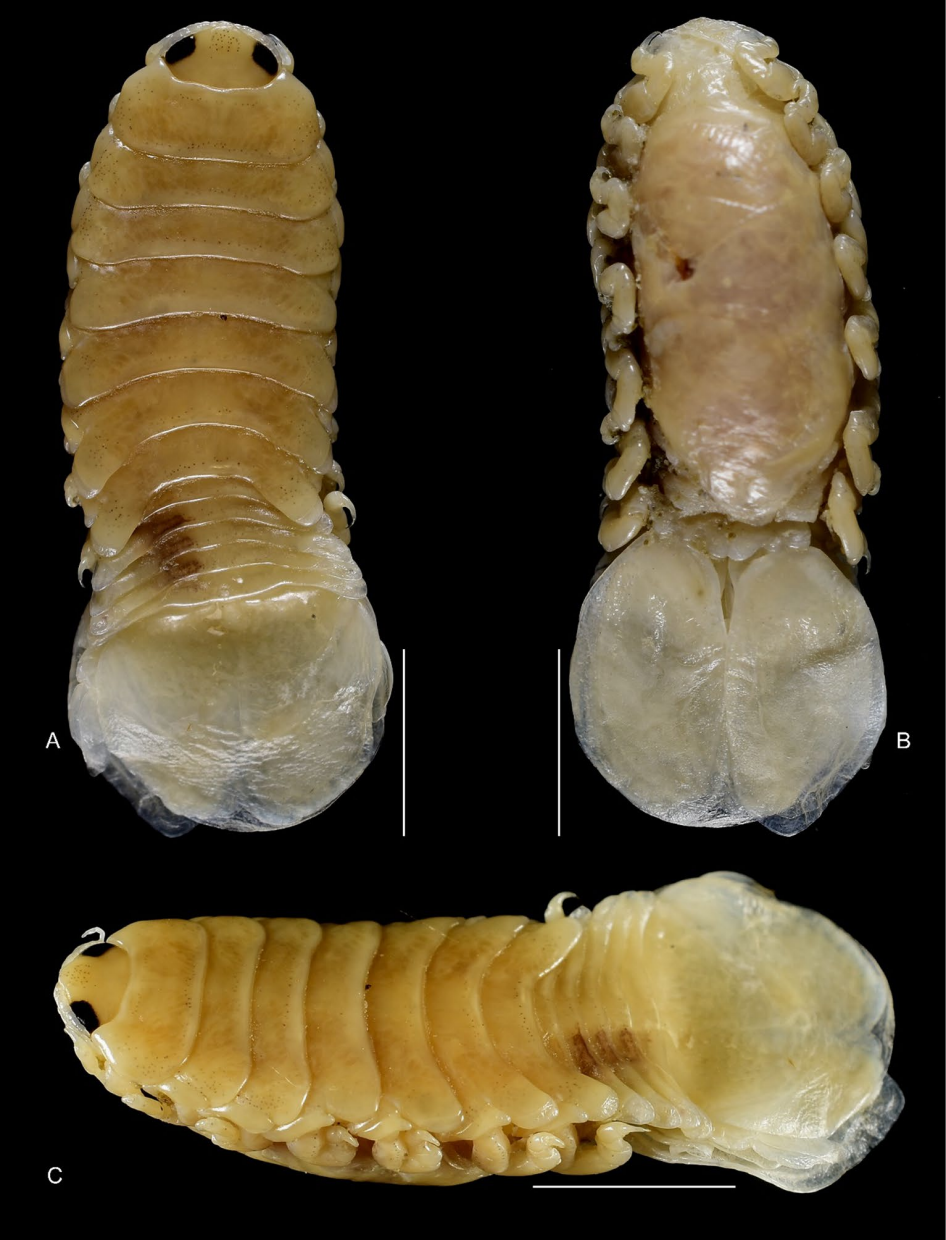
*Brucethoa isro* **n. sp.,** ovigerous female paratype (Reg. No. ZSI/WGRC /IR.INV./ 26316). A, dorsal view; B, ventral view; C, dorso-lateral view. *Scale, A – C: 5 mm.*

**Fig. 6:**
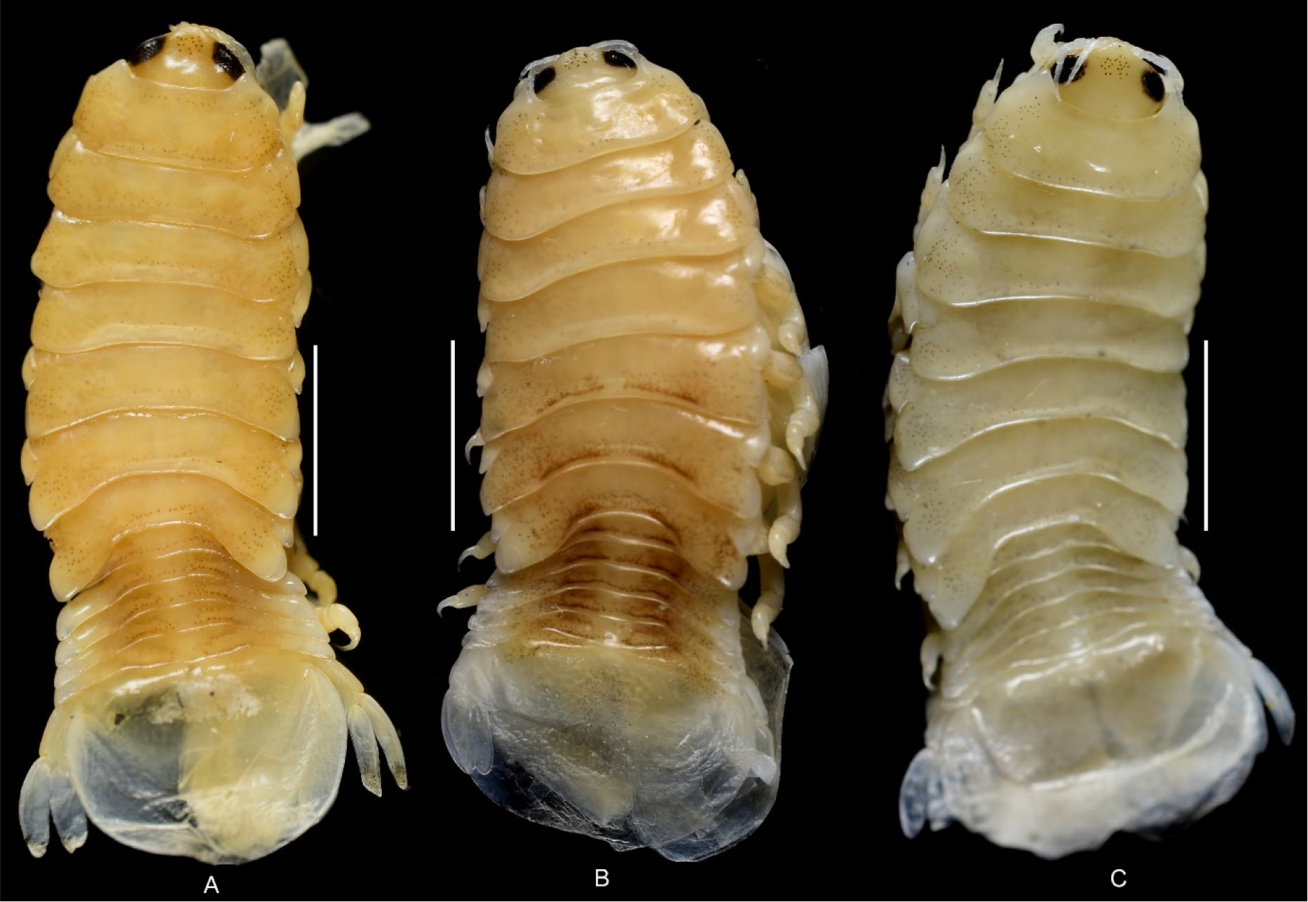
*Brucethoa isro* **n. sp.,** ovigerous females. A, paratype (Reg. No. CAH/INV/ISO 0303); B, paratype (Reg. No. ZSI/WGRC/IR. INV/26316); C, non-type (Reg. No. CAH/INV/ISO 0304). *Scale, A – C: 4 mm.*

**Fig. 7:**
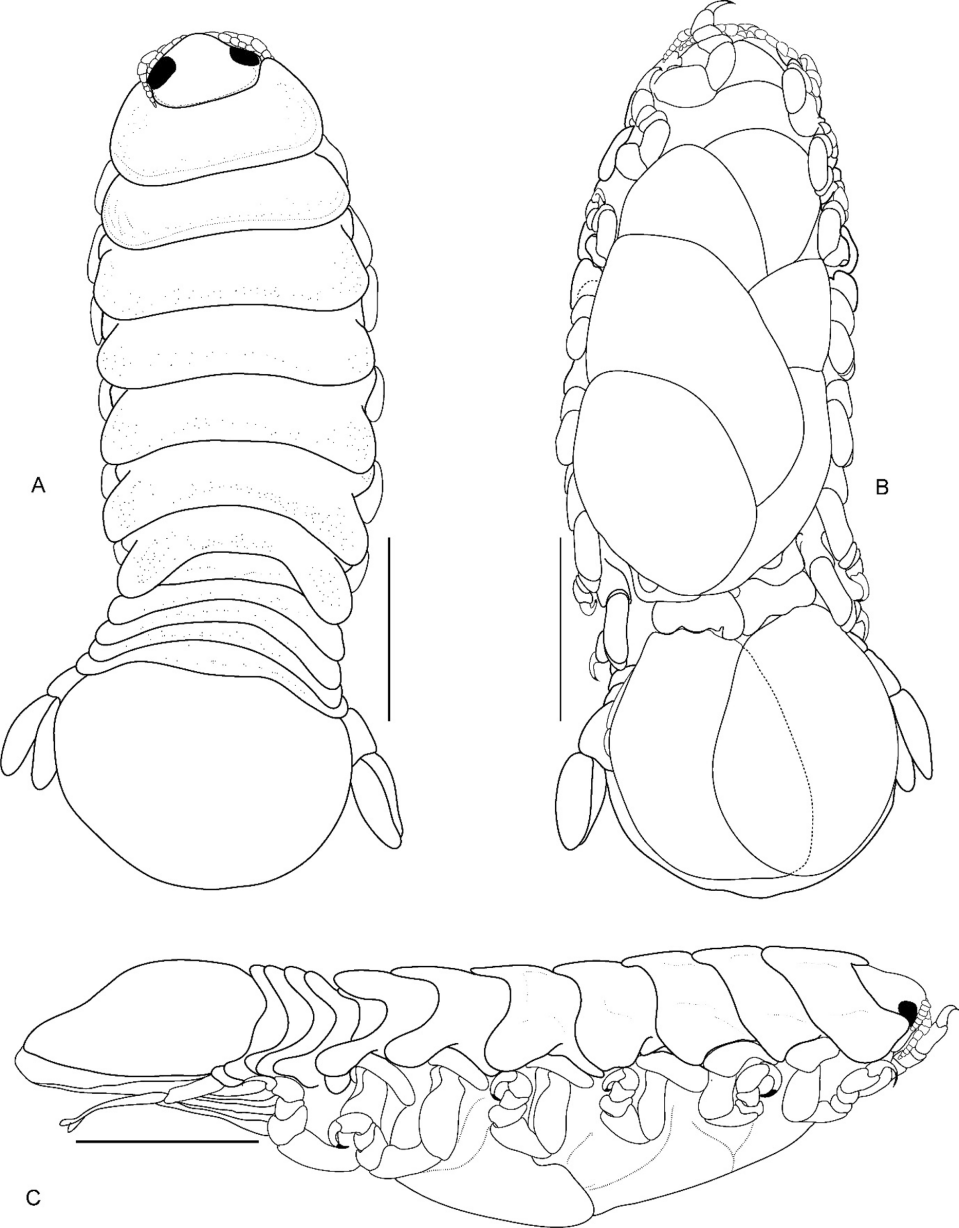
*Brucethoa isro* **n. sp.,** ovigerous female holotype (Reg. No. ZSI/WGRC/IR. INV/26312). A, dorsal view; B, ventral view; C, lateral view. *Scale: A-C: 5 mm.*

**Fig. 8:**
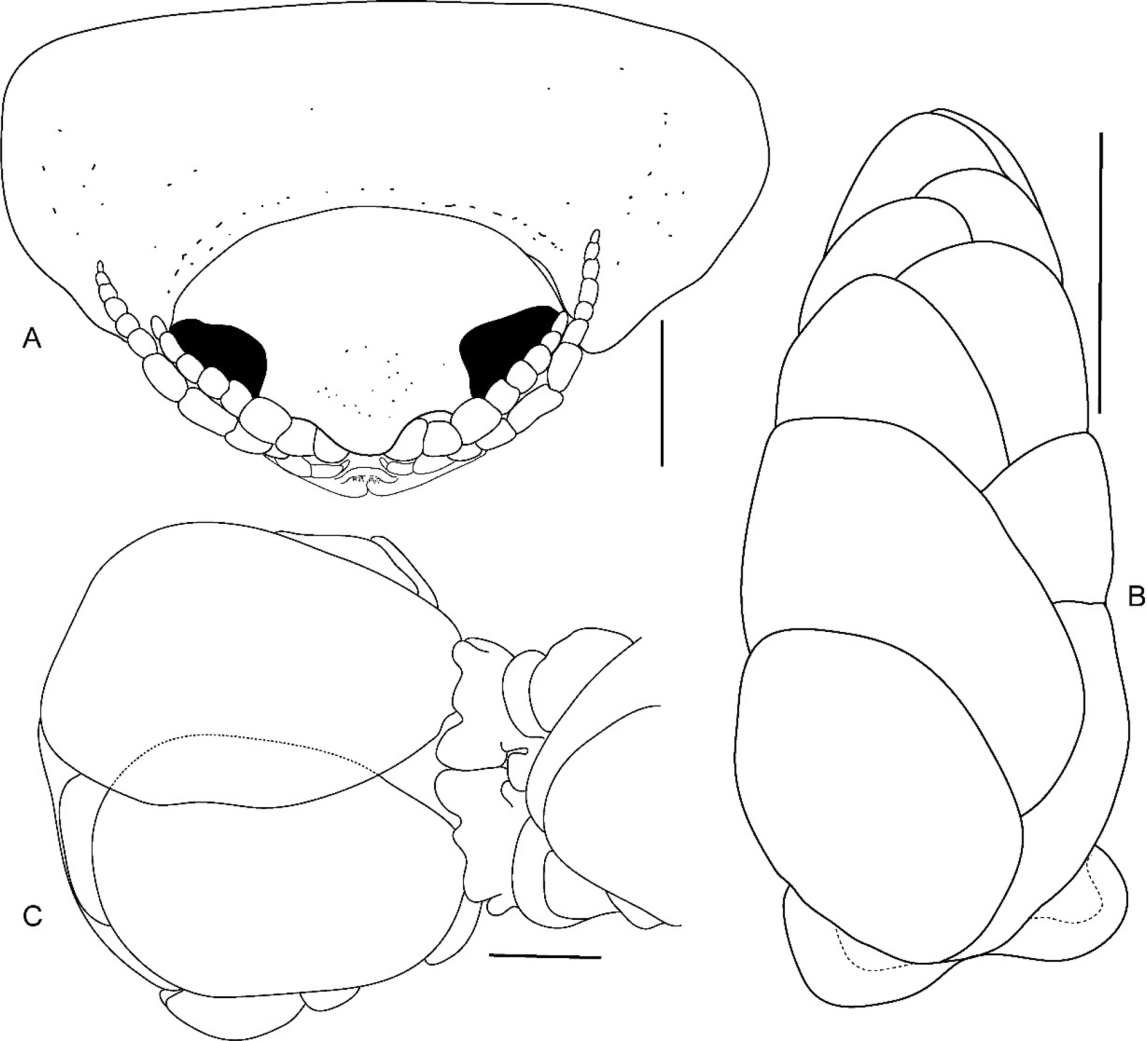
*Brucethoa isro* **n. sp.,** ovigerous female holotype (Reg. No. ZSI/WGRC/IR. INV/26312). A, cephalon dorso-frontal view; B, brood pouch with posterior lobes; C, ventral view of posterior pereon and pleon showing large posterior lobes on sternite of pereonite 7. *Scale: A:1 mm; B: 5 mm; C: 2 mm.*

**Fig. 9:**
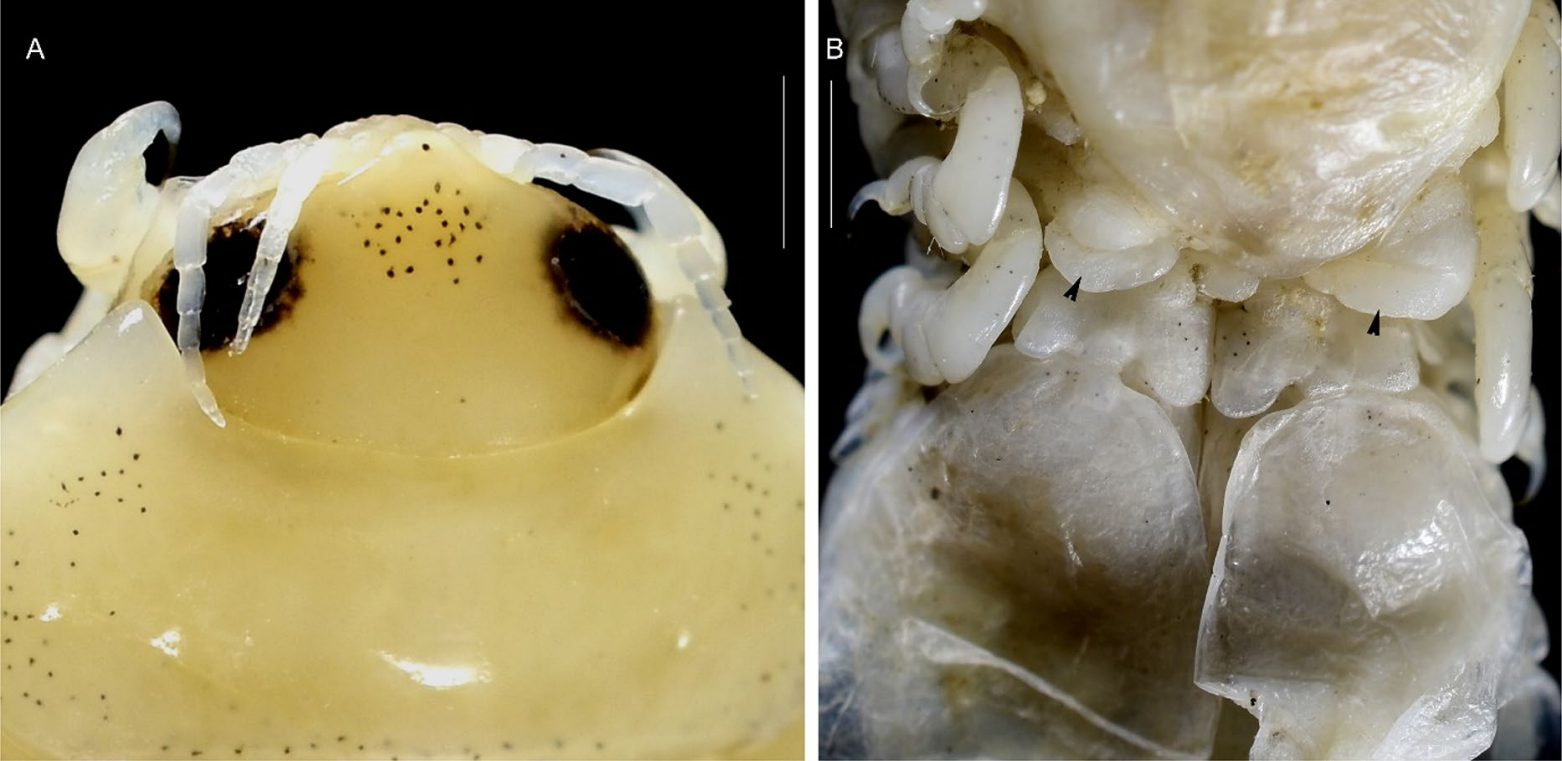
*Brucethoa isro* **n. sp.,** ovigerous female holotype (Reg. No. ZSI/WGRC/IR. INV/26312). A, cephalon dorso-frontal view; B, ventral view of posterior pereon and pleon showing large posterior lobes on sternite of pereonite 7. *Scale, A – B: 1 mm.*

**Fig. 10:**
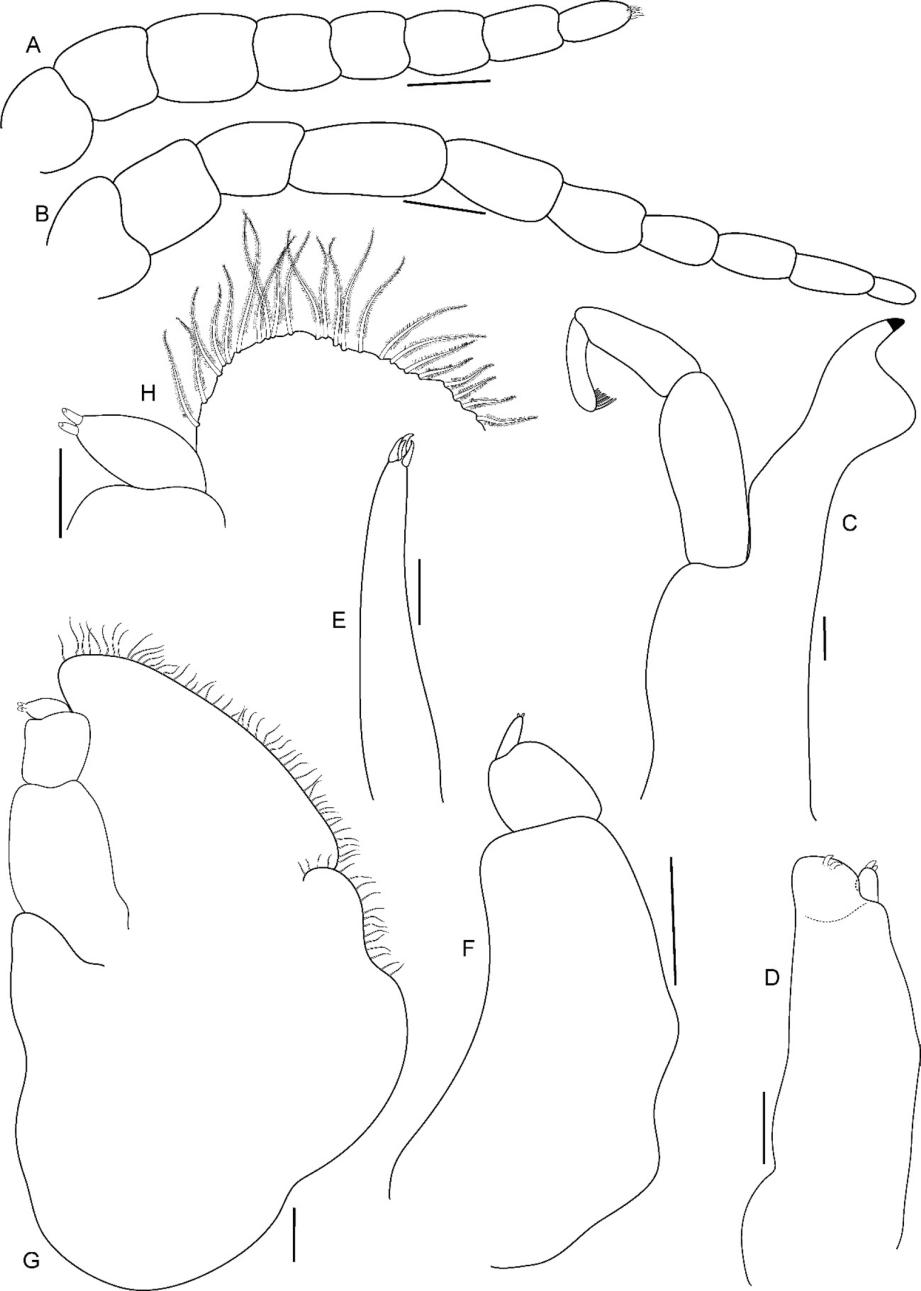
*Brucethoa isro* **n. sp.,** ovigerous female paratype (partially dissected) (Reg. No. ZSI/WGRC/IR. INV/26313). A, antennula; B, antenna; C, mandible; D, maxilla; E, maxillula; F, maxilliped of non-ovigerous female (Reg. No. ZSI/WGRC/ IR.INV./26315); G, maxilliped of ovigerous female; H, distal segment of maxilliped palp and plumose setae of maxilliped. *Scale: A, B, G: 0.2 mm; C-F, H: 0.1 mm*

**Fig. 11:**
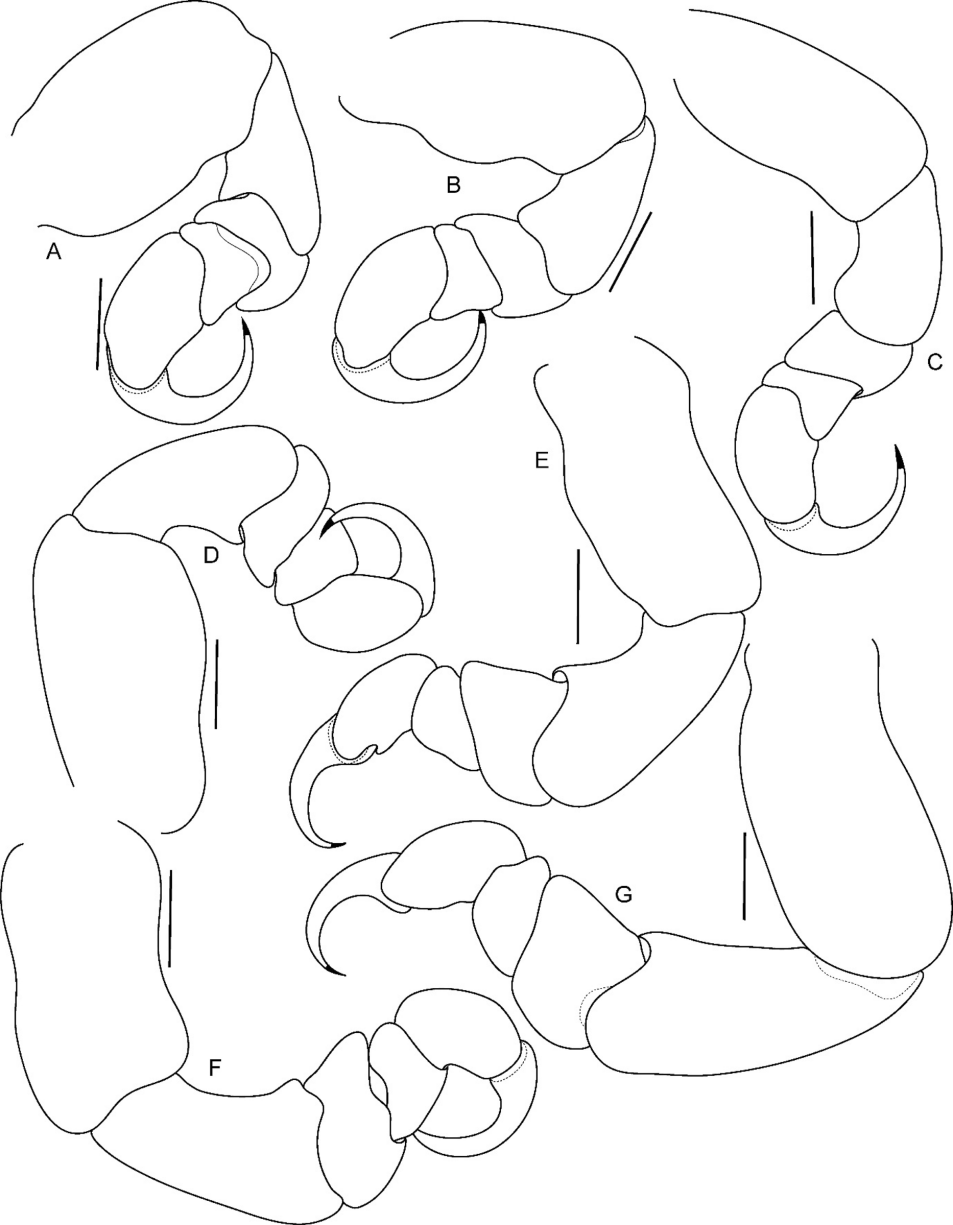
*Brucethoa isro* **n. sp.,** ovigerous female paratype (partially dissected) (Reg. No. ZSI/WGRC/IR. INV/26313). A–G, pereopods 1–7. *Scale: A-G: 0.5 mm*

**Fig. 12:**
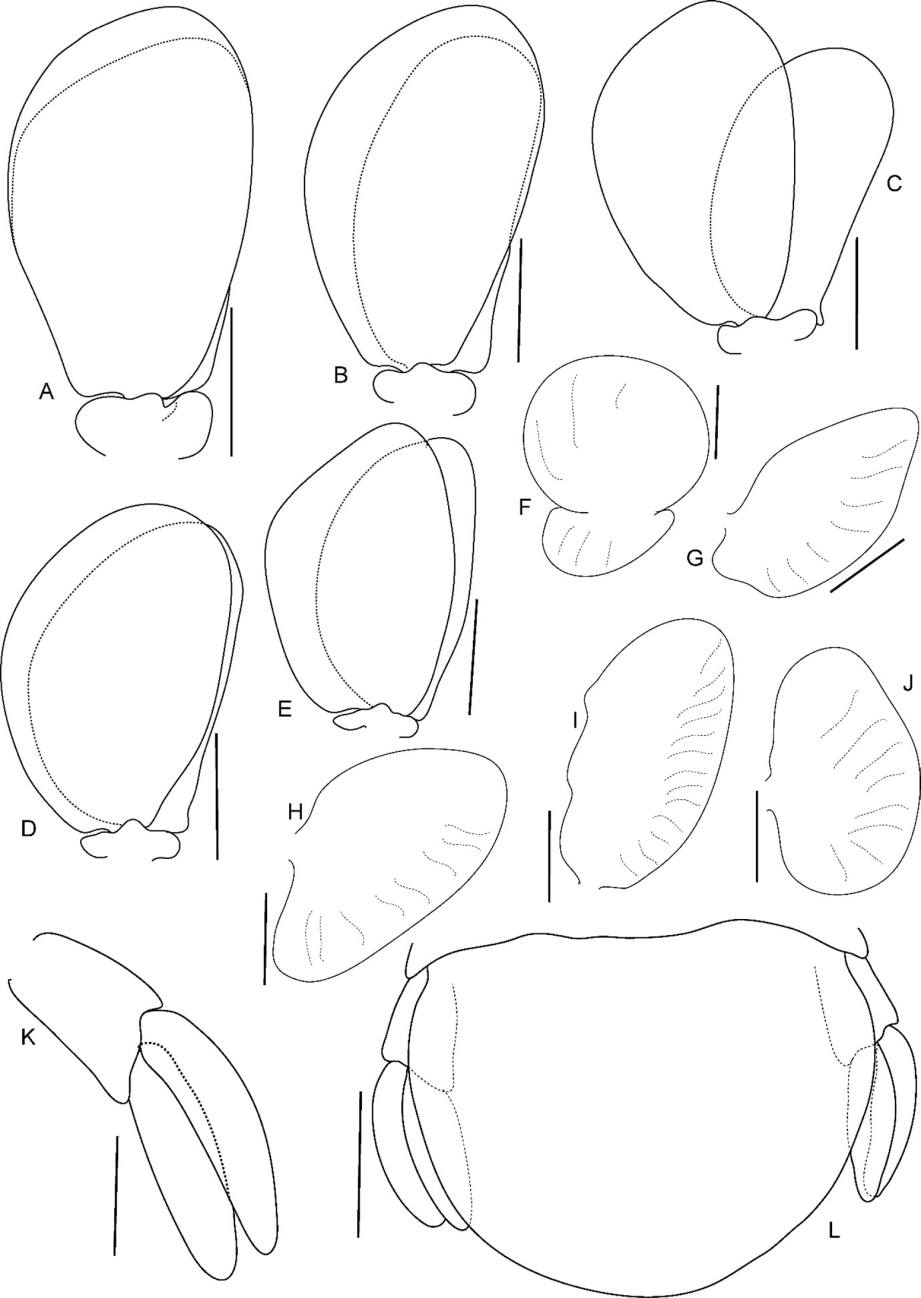
*Brucethoa isro* **n. sp.,** ovigerous female paratype (partially dissected) (Reg. No. ZSI/WGRC/IR. INV/26313). A–E, pleopods 1–5; F-J, brood plate (endopodite) of pereopod 1–5; K, uropod; L, pleotelson and uropods. *Scale: A-J, L: 2 mm; K: 1mm.*

**Fig. 13:**
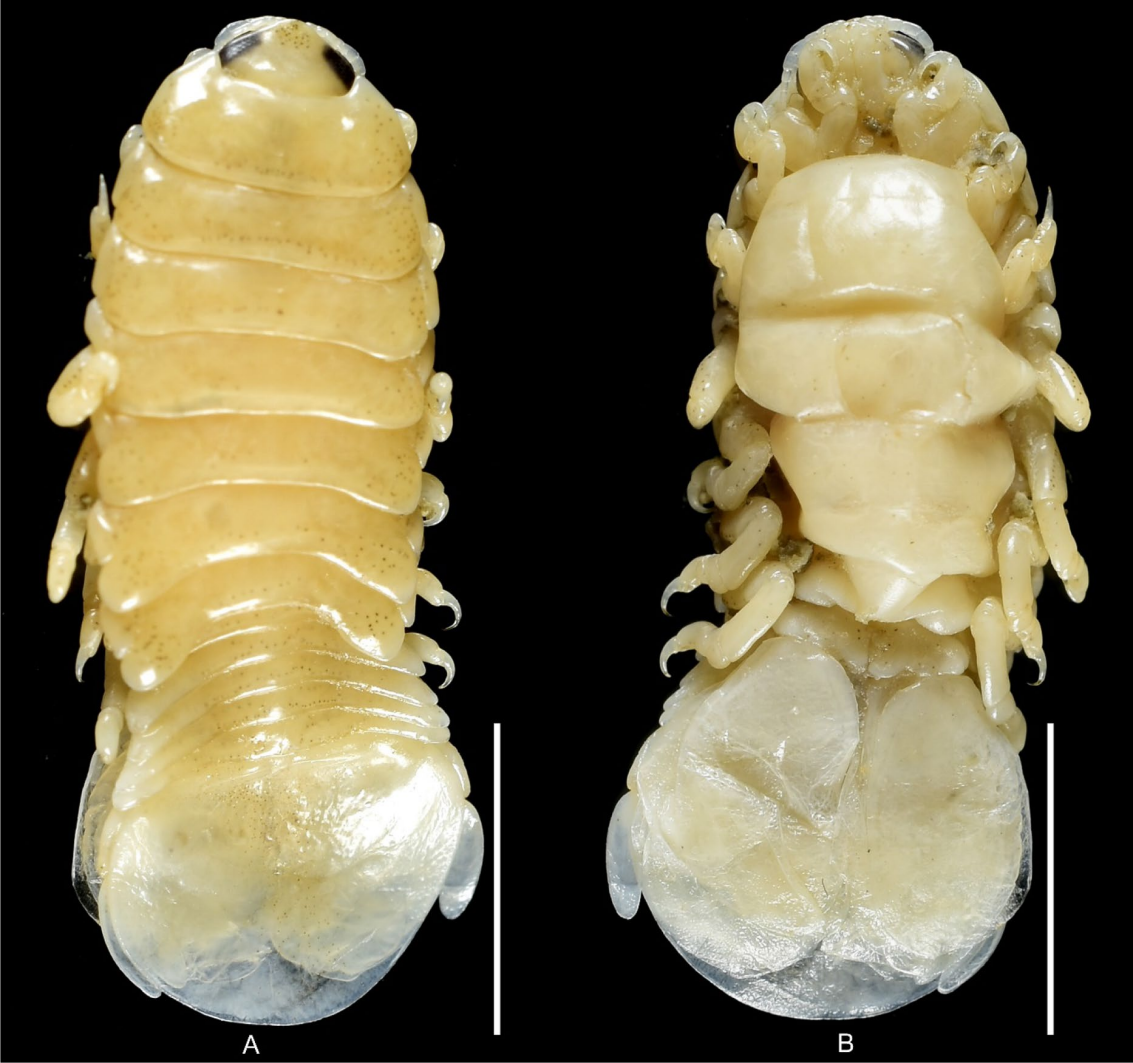
*Brucethoa isro* **n. sp.,** late transitional, A, dorsal view; B, ventral view. *Scale, A – B: 5 mm.*

**Fig. 14:**
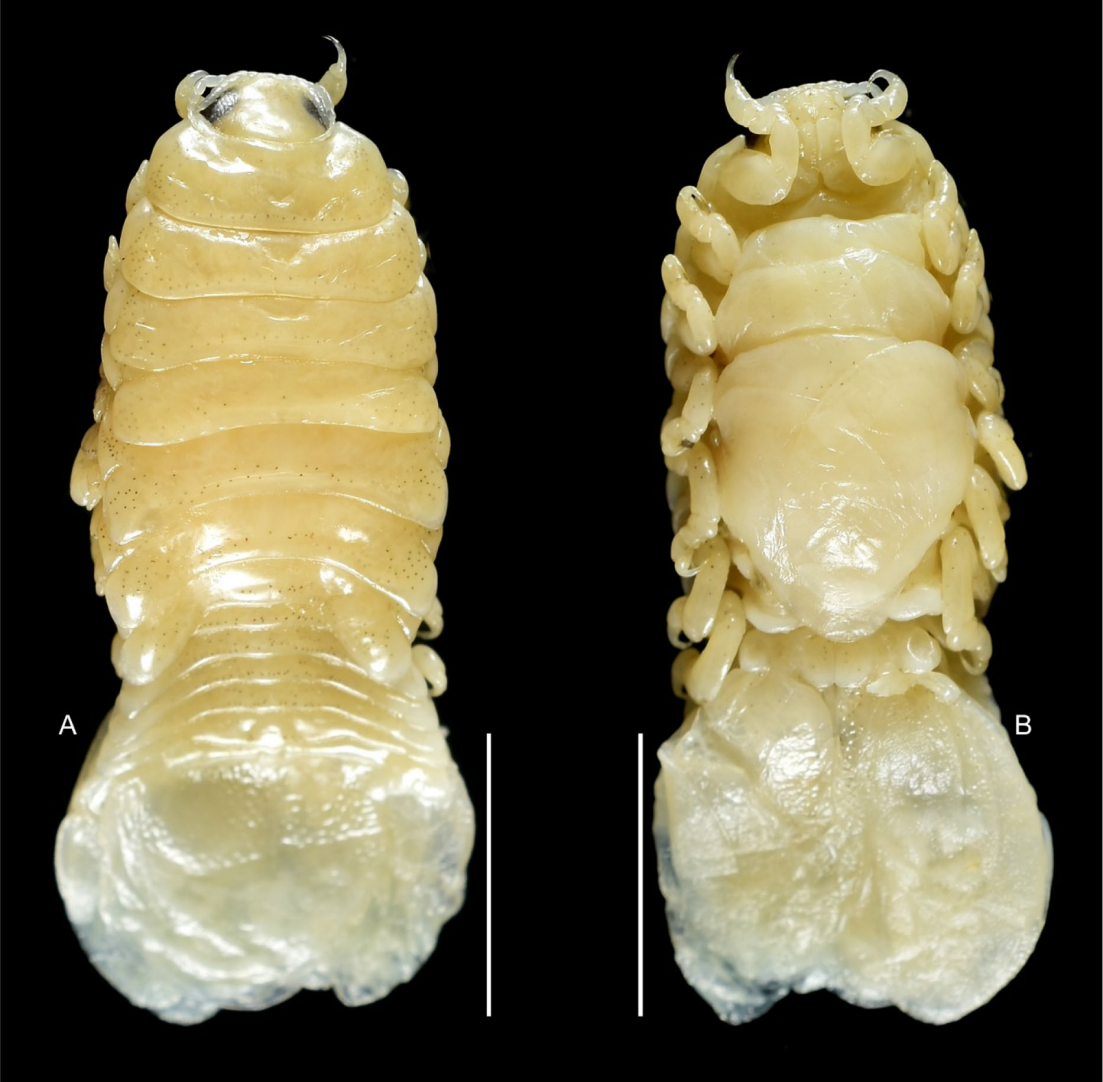
*Brucethoa isro* **n. sp.,** late transitional on moulting-transitional to female moult. A, dorsal view; B, ventral view. *Scale, A – B: 5 mm.*

**Fig. 15.**
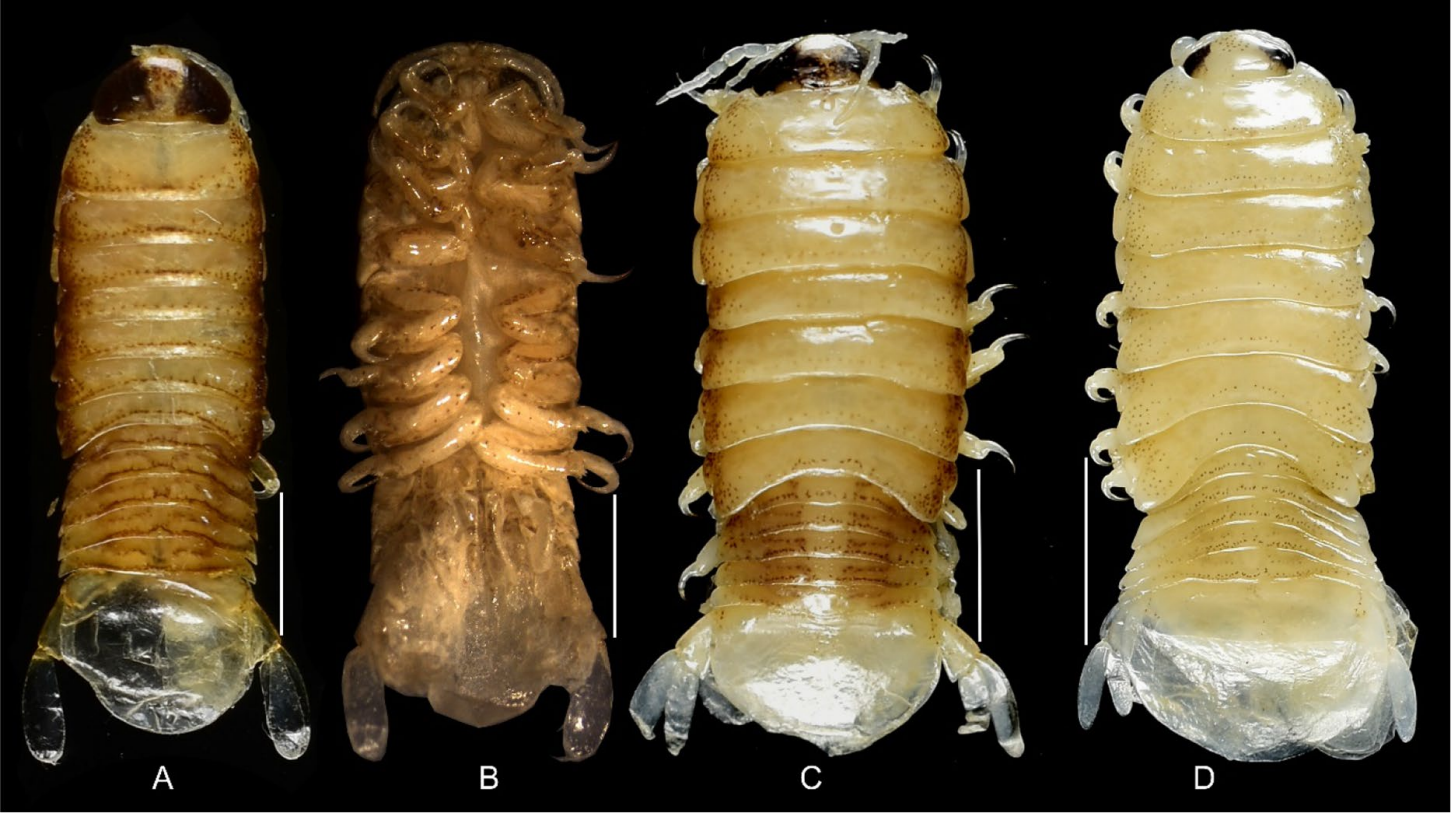
*Brucethoa isro* **n. sp.,** A. Juvenile (8.9 mmL_2.85mmW); B, Ventral view; C, Male (8.8 mmL_3.3mm W); D, early transitional (12mmL_4.2mmW). *Scale, A – C: 2 mm; D: 3 mm.*

**Fig. 16:**
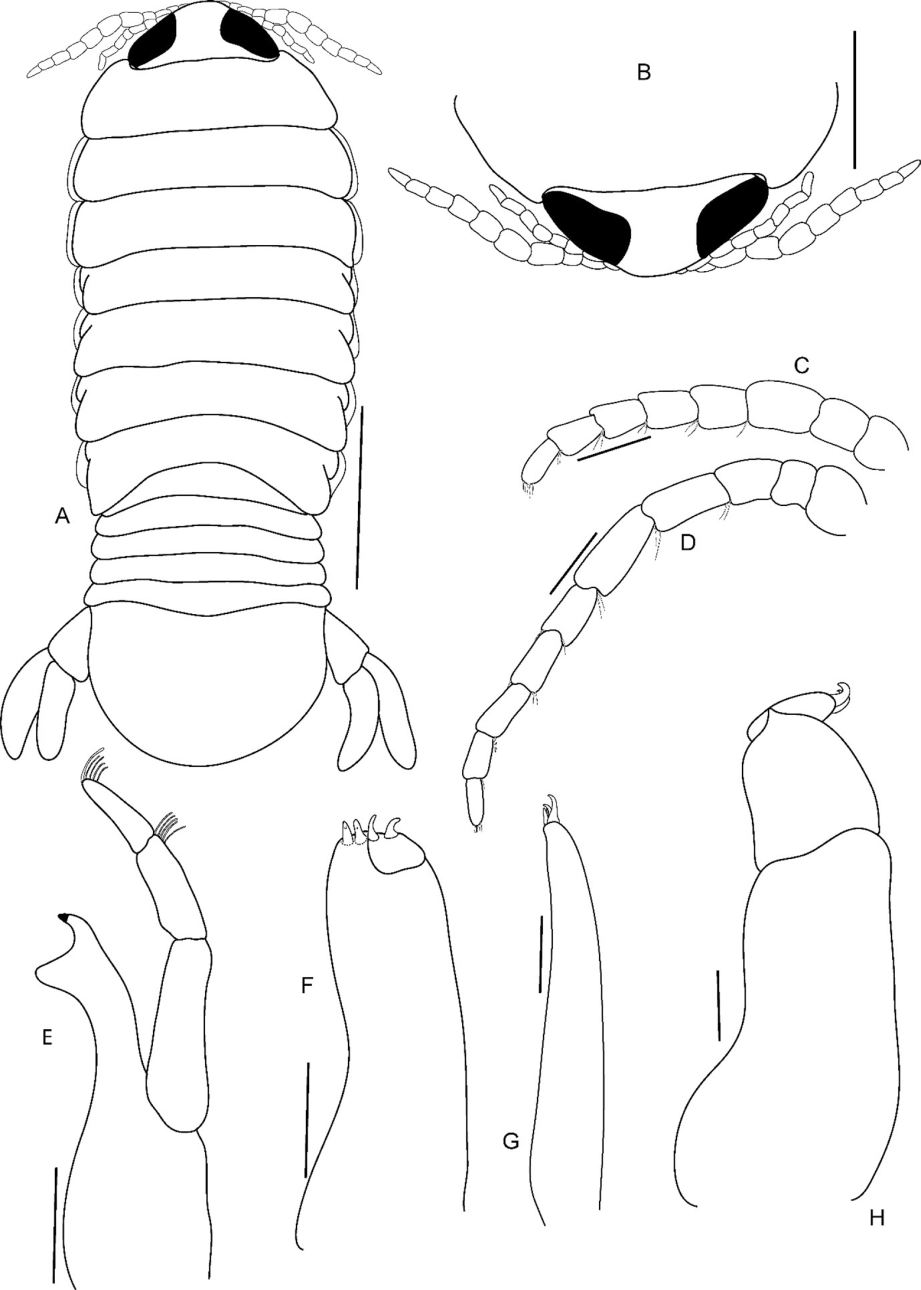
*Brucethoa isro* **n. sp.,** male (Reg. No. ZSI/WGRC/IR. INV/26319), A, dorsal view; B, cephalon dorsal view; C, antennula; D, antenna; E, mandible; F, maxilla; G, maxillule; H, maxilliped. *Scale: A: 2 mm; B: 1 mm; C- D: 0.5 mm, E: 0.3 mm; F-G: 0.2 mm; H: 0.1 mm.*

**Fig. 17:**
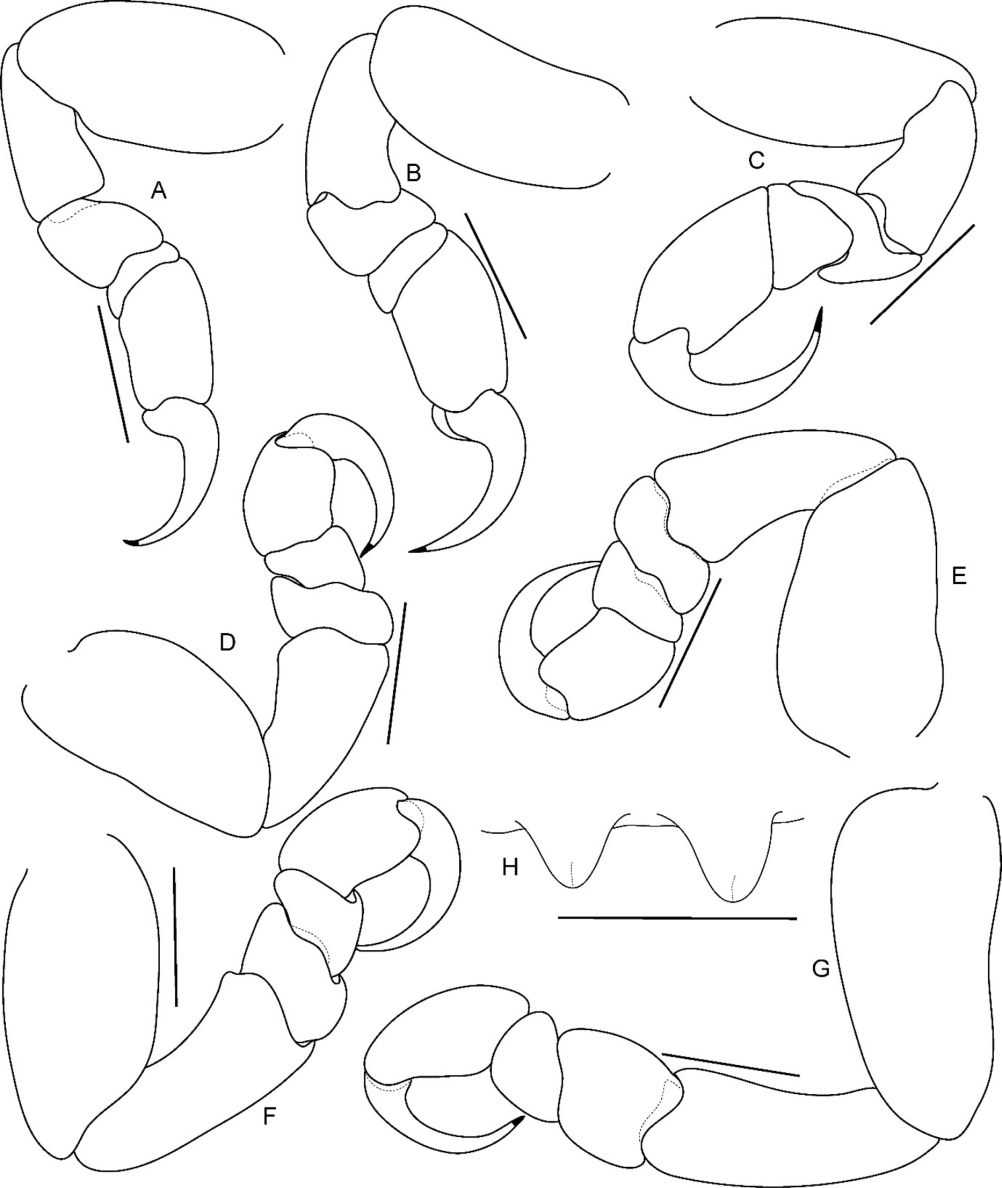
*Brucethoa isro* **n. sp.,** male (Reg. No. ZSI/WGRC/IR. INV/26319), A-G, pereopods 1–7; H, penis. *Scale: A-H: 0.5 mm*

**Fig. 18:**
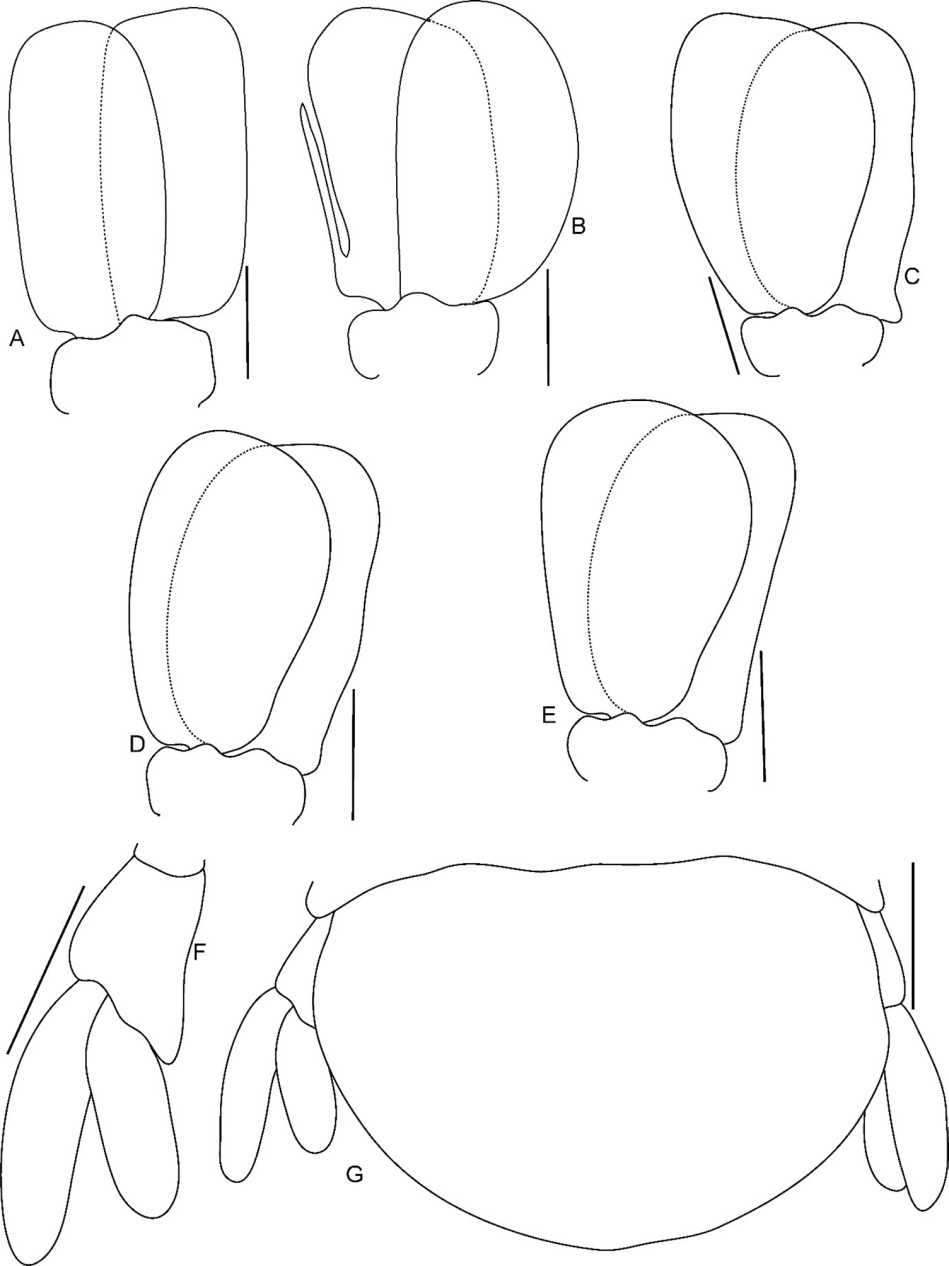
*Brucethoa isro* **n. sp.,** male (Reg. No. ZSI/WGRC/IR. INV/26319). A-E, pleopods 1–5; F, uropod; G, uropods, and pleotelson. *Scale: A-E: 0.5 mm; F-G: 1 mm*

**Fig. 19:**
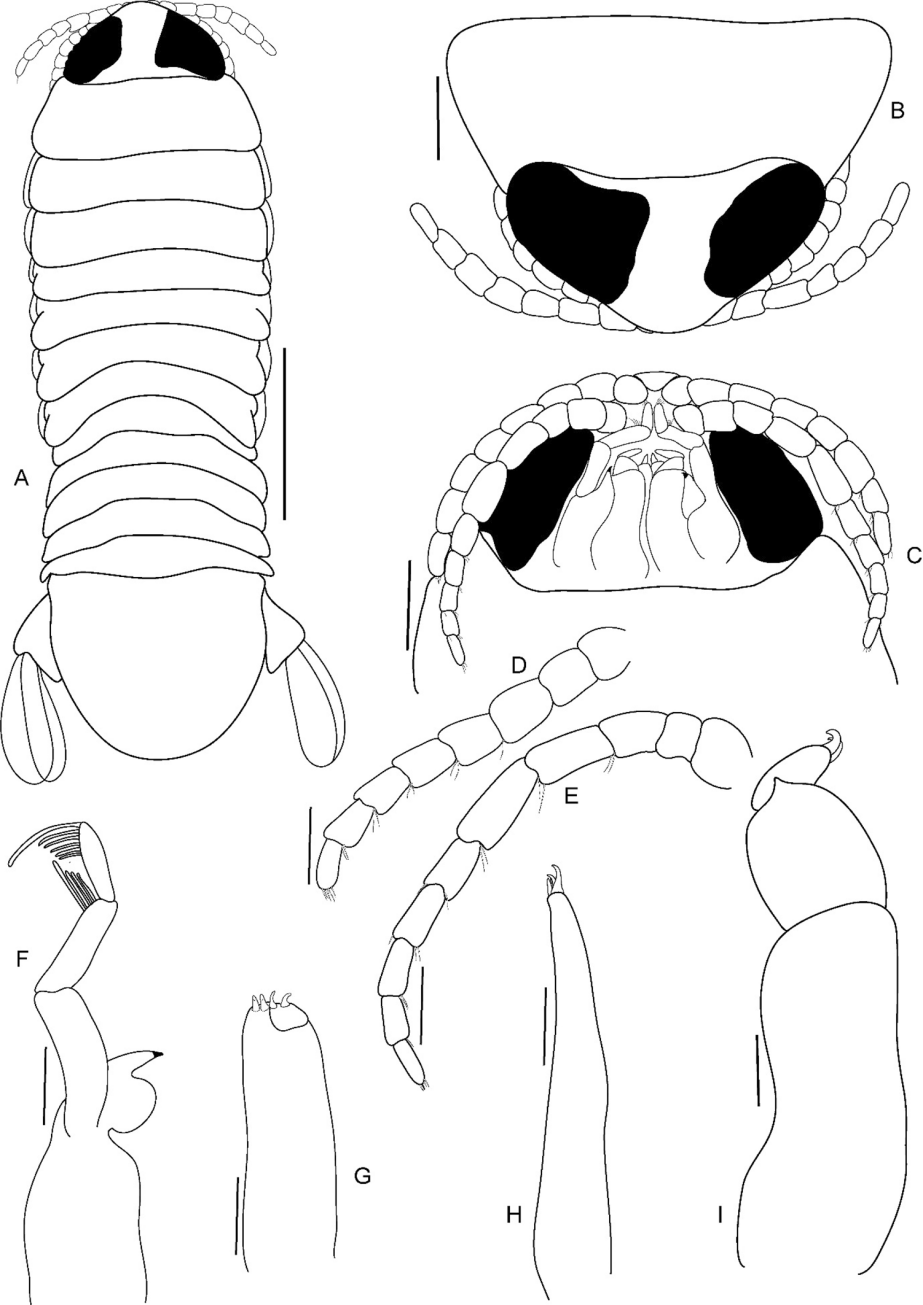
*Brucethoa isro* **n. sp.,** juvenile (Reg. No. ZSI/WGRC/IR. INV/26318), A, dorsal view; B, cephalon dorsal view; C, cephalon ventral view; D, antennula; E, antenna; F, mandible; G, maxilla; H, maxillule; I, maxilliped. *Scale: A: 2 mm; B-E: 0.5 mm; F-I: 0.1 mm*

**Fig. 20:**
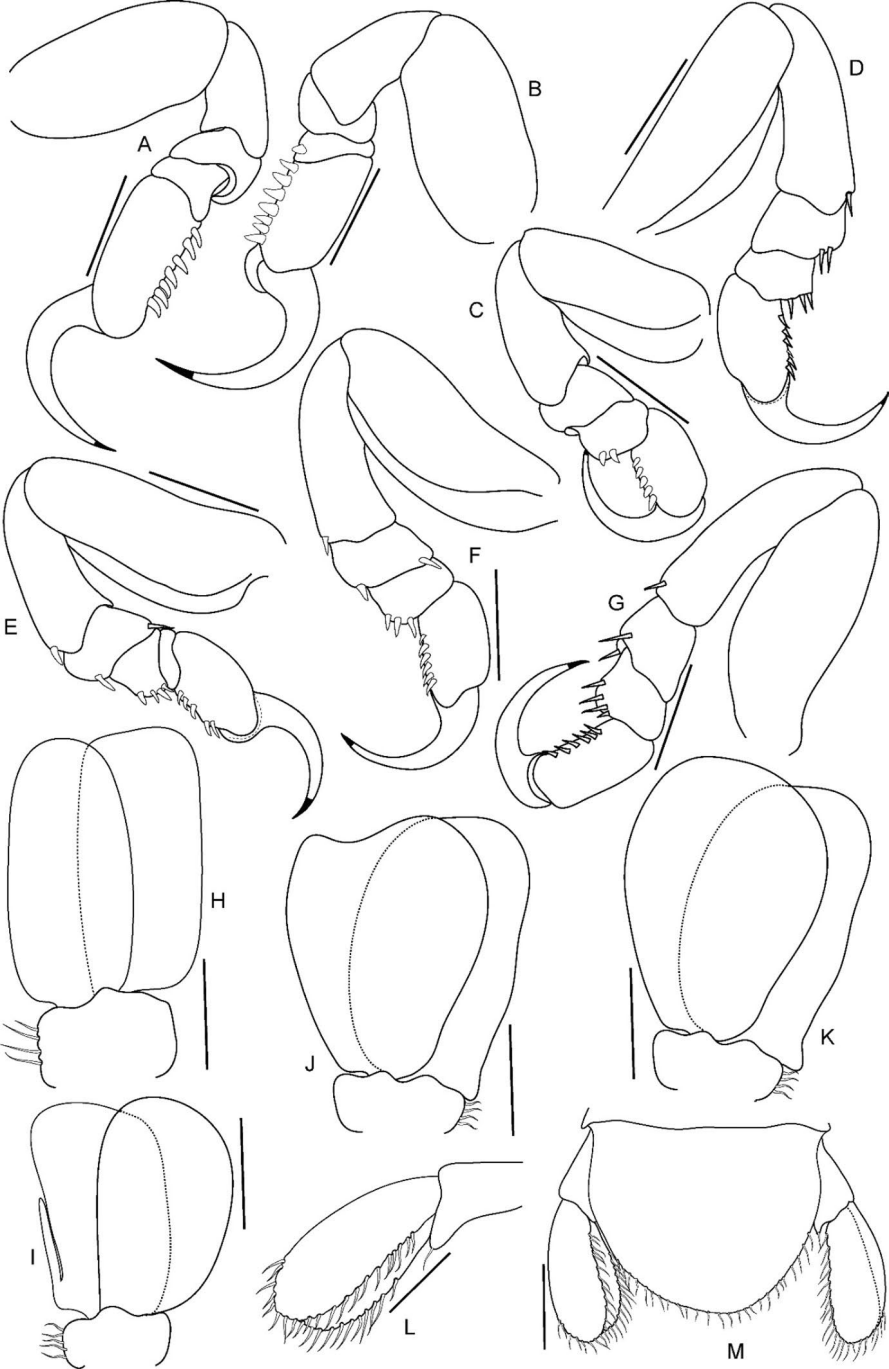
*Brucethoa isro* **n. sp.,** juvenile (Reg. No. ZSI/WGRC/IR. INV/26318). A–G, pereopods 1–7; H-I, pleopods 1–2; J-K, pleopods 4–5; L, uropod; M, uropods and pleotelson. *Scale: A-L: 0.5 mm; M: 1 mm*

***Material examined:***

*Type material: ****Holotype.*** 1 female [23.5 mm L, 7.0 mm W (maximum width) ovigerous], from Spinyjaw greeneye, *Chlorophthalmus corniger* Alcock, recorded from ~ 265 to 458 m depth, Neendakara (08^0^30.0'N 76^0^53.30'E), Kollam district, Kerala state, southwest coast of India, December 2018, coll. PT Aneesh (Reg. No. ZSI/WGRC/IR.INV./26312). ***Paratypes:*** Same data as holotype with the following measurements and registration details: 1 female [19.0 mm L, 6.0 mm W, ovigerous, partially dissected], (Reg. No. ZSI/WGRC /IR.INV./26313); 1 female [21.5 mm L, 8.0 mm W, ovigerous], (Reg. No. ZSI/WGRC/IR.INV./26314); 1 female [19.0 mm L, 7.0 mm W, non-ovigerous], (Reg. No. ZSI/WGRC/IR.INV./26315); 1 female [16.0 mm L, 6.2 mm W, non-ovigerous on molting (oostigition molt)], (Reg. No. CAH/INV/ISO 0302); 1 female [19.5 mm L, 7.3 mm W, ovigerous], (Reg. No. ZSI/WGRC/IR.INV./26316); 1 female [20.0 mm L, 7.5 mm W, ovigerous], (Reg. No. CAH/INV/ISO 0303); 1 female [19.5 mm L, 7.3 mm W, ovigerous], (Reg. No. CAH/INV/ISO 0304); 1 late transitional [12.3 mm L, 4.0 mm W, ovigerous], (Reg. No. ZSI/WGRC/IR.INV./26317); 1 juvenile [8.9 mm L, 2.8 mm W, partially dissected], (Reg. No. ZSI/WGRC/IR.INV./26318); 1 male [8.8 mm L, 3.3 mm W, partially dissected], (Reg. No. ZSI/WGRC/IR.INV./26319); 1 early transitional [12 mm L, 4.2 mm W, partially dissected], (Reg. No. CAH/INV/ISO 0305).

***Description of female (***Figs 1–12*). Body* elongated 2.9*–*3.15 times as long as greatest width, dorsal surfaces weakly or not vaulted, almost flat, widest at pereonite 4*–*5, most narrow at pereonite 1. *Cephalon* 1.6 times wider than long, moderately immersed in pereonite 1. *Frontal margin* with acute ventrally directed rostral point. *Eyes* oval with distinct margins, one eye 0.3 times width of cephalon. Pereonite 1 smooth; anterior border medially straight, slightly curved laterally; anterolateral angles with a small distinct produced point. *Coxae* narrow, reniform, shorter than respective pereonite; 0.5 times pereonite length; all visible in dorsal view. *Pereonites* 1–4, posterolateral angles not produced, 5–7 slightly produced. Pereonite 7 posterolateral margins extending posteriorly to pleonite 1. Pereonites slightly increase the width from 1–3; pereonites 4–5 subequal in width. Pereonite 1 longest; 3–6 subequal; 7 shortest. *Pleon* short, ~16% BL, pleonites all visible, pleon wide, 0.95 as wide as pereon max. width; pleonite 1 narrower than others; pleonites progressively increasing in width towards posterior, with lateral gaps between pleonites; pleonites 1–4 medially subequal in length, pleonite 5 longest. *Pleotelson* 0.9 times as wide as pereonite 7; lateral margin convex, not folded; posterior margin, broadly rounded, 1.3 times as wide as long.

*Antennula* shorter than antenna, with eight articles, narrowly separated by rostrum; terminal article with few short simple setae. *Antenna* slender, with ten articles; article 4 longest; terminal article shortest, extending to anterior margin of pereonite 1. Buccal cone positioned strongly to anterior, overriding and obscuring antennal bases, visible in frontal view. *Mandible* with molar process, palp articles all slender, article 1, twice the length of article 3; palp article 3 with 9, simple setae. Maxillula with four acuminate terminal RS. Maxilla mesial lobe distinct, with two acute apical RS, lateral lobe with two acute RS. *Maxilliped* article 3 with two recurved RS. Maxilliped with oostegital lobes, lateral margins with many plumose setae; mouthparts not covered by oostegites of pereopod 1.

*Pereopods* articles not dilated or expanded; dactyli relatively short, strongly curved. *Pereopod 1*, basis 1.6 times as long as greatest width; ischium 0.7 times as long as basis; merus 0.55 times as long as wide; propodus 1.6 times as long as wide; dactylus 1.25 times as long as propodus, 3.3 times as long as proximal width. *Pereopod 2* basis twice as long as greatest width; ischium 0.6 times as long as basis; propodus 1.8 times as long as wide; dactylus 3.4 times as long as proximal width. *Pereopod 3* basis 2.1 times as long as greatest width; ischium 0.65 times as long as basis, 1.8 times as long as wide; propodus 1.6 times as long as wide; dactylus, 1.4 times as long as propodus, 3.2 times as long as proximal width. *Pereopod 4* basis 1.6 times as long as greatest width; ischium 0.85 times as long as basis, 1.9 times as long as wide; propodus 1.6 times as long as wide; dactylus, 1.4 times as long as propodus, 3.1 times as long as proximal width. *Pereopod 5* basis twice as long as greatest width; ischium 0.85 times as long as basis, 1.8 times as long as wide; propodus twice as long as wide; dactylus, 1.2 times as long as propodus, 3.0 times as long as proximal width. *Pereopod 6* basis 1.7 times as long as greatest width, ischium 0.9 times as long as basis, 1.7 times as long as greatest width; merus 0.5 times as long as wide; carpus 0.8 times as long as wide; propodus 1.8 times as long as wide; dactylus 1.35 times as long as propodus, 3.0 times as long as greatest width. *Pereopod 7* slightly longer than pereopod 6; basis 1.9 tims as long as greatest width; ischium as long as basis, 2.5 times as long as wide; merus 0.85 as long as wide, 0.35 times as long as ischium; carpus 0.6 times as long as wide, 0.2 times as long as ischium; propodus 1.8 times as long as wide, 0.4 times as long as ischium; dactylus 1.2 times as long as propodus, three times as long as basal width.

Brood pouch from coxae 1–4 and 6, proximally thick; posterior pocket formed by two prominent fleshy lobes.

*Pleopods* large, slightly visible in dorsal view, extending laterally and posteriorly slightly beyond pleotelson margins; rami all simple, without proximomedial lamellar lobe, folds or thickened ridges. Pleopod peduncle lateral lobes absent.

*Pleopod 1* exopod 1.6 times as long as wide, lateral margin convex, distally broadly rounded, mesial margin convex; endopod 0.85 as long as exopod, 1.4 times as long as wide, lateral margin weakly convex, distally broadly rounded; peduncle twice as wide as long. *Pleopod 2* exopod 1.55 times as long as wide, lateral margin convex; endopod 0.8 as long as exopod, 2.1 times as long as wide. *Pleopod 3* exopod 1.6 times as long as wide; endopod 0.9 as long as exopod, 1.7 times as long as wide. *Pleopod 5* exopod 1.5 times as long as wide, lateral margin convex; endopod 0.9 as long as exopod, 1.7 times as long as wide. Pleopods decreasing size from 1–5.

*Uropod* 0.8 times as long as pleotelson; peduncle 0.6 times as long as endopod, marginal setae absent. *Endopod* 3.8 times as long as greatest width, 0.85 times as long as exopod, lateral margin convex. *Exopod* curved to mesial, 4.0 times as long as greatest width, mesial margin concave, lateral margin convex.

***Description of transitional stage (***Figs. 13–14*). Body* 2.9*–*3.1 times longer than wide; dorsal surfaces not vaulted, widest at pereonite 4–5, most narrow at pereonite 1. *Cephalon* similar to that of the female. Pereonites and pleonites of the late stage similar to those of the female. Antennula, antenna, mandible palp, maxilla, maxilliped similar to those of the male. Coxae similar to that of the female. Penial processes rudimentary. Pleotelson 1.3 times wider than long. Uropods similar to those of male. Pereopods and pleopods more similar to those of male in early stage, and those of late stage more similar to those of female.

***Description of male (***Figs 15c,d, 16–18*). Body* symmetrical, 2.5 times as long as greatest width, dorsal surfaces smooth, lateral margins sub-parallel, widest at pereonite 3, pereonite 2–6 subequal in width, most narrow at pereonite 7. *Cephalon* 2.6 times wider than long, anterior border slightly triangular. *Frontal margin* similar to that of female. *Eyes* conspicuous, one eye 0.3 times width of cephalon. *Coxae* all dorsally visible. *Pereonite*s 1–5 posterolateral angles not produced, pereonites 6–7 slightly produced. Pereonite 7 posterolateral margin slightly extending beyond the lateral margin of pleonite 1. *Pereonites* 3–6 more or less equal in width, 1 narrower than others. Pereonite 1 longest, 7 shortest. *Pleon* wide, 0.8 times as wide as pereon; pleonites 2–4 more or less equal in width, 1 narrower than others, 5 widest. *Pleotelson* 0.9 times as wide as pereonite 7; posterior margin broadly rounded, 1.5 times as wide as long.

*Antennula* length shorter than antenna, eight articled; articles 3–8 with few setae. *Antenna* with ten articles; article 5 longer than others; articles 3–10 with few setae. *Mandible* with molar process, palp articles all slender, article 1, 2.1 times longer than article 3; palp article 2 with five, article 3 with nine, simple setae. Maxillula with four acuminate terminal RS. Maxilla similar to that of non-ovigerous female. *Maxilliped* article 3 with two recurved RS.

*Pereopods*, without carina, without spines or setae*. Pereopod 1* basis 1.8 times as long as greatest width; ischium 0.6 times as long as basis; merus as long as wide; propodus 1.75 times as long as wide; dactylus, 1.2 times as long as propodus, 2.8 times as long as proximal width. *Pereopod* 2 basis 2.1 times as long as greatest width; propodus 1.4 times as long as wide, 0.55 as long as basis; dactylus, 1.4 times as long as propodus. *Pereopod* 3 basis twice as long as greatest width; propodus 1.7 times as long as wide, 0.7 as long as basis; dactylus, 1.1 times as long as propodus. *Pereopod* 4 basis 1.85 times as long as greatest width; propodus 1.3 times as long as wide, 0.4 as long as basis; dactylus, 1.65 times as long as propodus. *Pereopod* 5 basis 1.6 times as long as greatest width; propodus 1.4 times as long as wide, 0.55 as long as basis; dactylus, 1.7 times as long as propodus. *Pereopod 6* basis twice as long as greatest width, ischium 0.8 times as long as basis; propodus 1.4 times as long as wide; dactylus 1.7 times as long as propodus. *Pereopod* 7 basis 2.1 times as long as greatest width; ischium 0.8 times as long as basis; merus as long as wide; propodus twice as long as wide; dactylus 1.1 times as long as propodus.

Penial process acute, twice as long as basal width, separated by 28 % width of sternite 7, visible on sternite 7, basally mutually adjacent.

Pleopods not extending beyond pleotelson margins, not visible in dorsal view. Pleopods 1–5 rami simple, without proximomedial lamellar lobe, folds or thickened ridges; endopod of all pleopods slightly shorter than exopod. *Pleopod 1* exopod 1.9 times as long as wide, distally rounded; endopod as long as exopod; peduncle 2.9 times as wide as long. *Pleopod* 2 appendix masculina straight, narrow, 0.7 as long as endopod. *Pleopod 5* exopod 1.7 times as long as wide, distally rounded; endopod 0.9 times as long as exopod.

*Uropod* 0.85 as long as pleotelson; peduncle 0.6 times as long as exopod, lateral margin without setae; rami not reaching the distal margin of pleotelson, marginal setae absent, apices rounded. *Exopod* 1.3 times as long as endopod; 3.25 times as long as wide. *Eendopod* 2.9 times as long as greatest width.

***Description of juvenile (***Fig. 15a, b, 19–20*). Body* transparent, 3.0 times longer than wide. *Eyes* black, distinct, 0.6 times as long as cephalon, conspicuous in dorsal view. *Cephalon* 2.0 times wider than long. Pereonite 3 widest, 1 longest. Pereonite 7 shortest, 4–6 subequal in length. All pleonites visible subequal in length and width. Pleotelson 1.3 times wider than long; apical margin with 24–26 plumose setae.

Antennula with eight articles, extending up to the anterior margin of pereonite 1; articles 3–8 with 2–3 spinules. Antenna longer than antennula, with ten articles, extends beyond the anterior margin of pereonite 1; articles 3–10 with 2–4 spinules. Mandible palp article 3 with one long and five short marginal setae; article 2 with four unequal setae. Maxillula, maxilla, and maxilliped similar to those of the male stage.

All pereopods with acute robust seta/e (RS). *Pereopods*, without carina. *Pereopod 1* basis 2.1 times as long as greatest width; ischium 0.5 times as long as basis; propodus longer than ischium, inner lateral margin with 7 RS. The setal formula of the pereopods is represented in the following table.

**Table:** 

	Basis	Ischium	Merus	Carpus	Propodus	Dactylus
Pereopod 1	0	0	0	0	7	0
Pereopod 2	0	0	0	1	7	0
Pereopod 3	0	0	0	2	5	0
Pereopod 4	0	1	2	3	6	0
Pereopod 5	0	1	2	4	5	0
Pereopod 6	0	1	2	3	6	0
Pereopod 7	0	1	2	4	6	0

Pleopods 1–5 peduncle with four spinules; endopod and exopod without setae. Uropod rami endopod as long as exopod, extending beyond the distal margin of pleotelson; peduncle with one apical seta. Exopod with 10–22 and endopod with 16–18 plumose setae.

***Body size:*** Ovigerous females (17–21.5 mm), non-ovigerous females (14–20 mm), males (8–9 mm), early transitional (8–12 mm), late transitional (14–16 mm), juvenile 8.9 mm.

***Colour:*** Body pale tan.

***Distribution:*** Neendakara, Kollam district, Kerala state, southwest coast of India (Type locality); Thoothukudi, Tamil Nadu, southwest coast, India.

***Host:*** Only known from the type host Spinyjaw greeneye, *Chlorophthalmus corniger* Alcock deep water depth range **~**219**–**800 meters.

***Etymology:*** The new specific name **‘isro**’, is an abbreviated name of the “Indian Space Research Organisation”, the national space agency of India, under the Department of Space, Government of India, which significantly contributed to many successful space missions, including the recent successful lunar mission titled “*Chandrayaan 3”.*

**Remarks**: *Brucethoa* *isro* **n. sp.** is identified by the following characters: head weakly immersed in pereonite 1, very elongated body (3.15 times as long as wide); body dorsum not vaulted, almost flat; all coxae short, 0.5 times as the length of corresponding pereonites; sternite 7 with prominent posterior lobes. *Brucethoa* *isro* **n. sp.** is the second species under the genus. All life stages of the new species are described [including females (ovigerous and non-ovigerous), males, transitional, and juvenile for precise identification irrespective of the life stages.

### Ecological remarks

From November 2017 to November 2021, along the southwest coast of India, we closely examined 52 species of deep-sea fishes for the presence of parasitic cymothoids. The cymothoid isopod, *Brucethoa* *isro* **n. sp.** was recovered only from spinyjaw greeneye, *Chlorophthalmus corniger* signifying its oligoxenous host specificity. A total of 76 individuals of *C. corniger* were examined from the available localities along the southwest coast of India. Of these, 37 individuals were infested with *B.* *isro* **n. sp.,** the prevalence being 48.6%. Twenty females (22 ovigerous and 8 non-ovigerous), 12 transitional stages (4 early and 3 late), 18 males, and two juveniles were recovered from these 37-infested host fish. Parasites were usually found in pairs in the host fish, one in each branchial cavity; mostly male-female pairs were found; the relatively large ovigerous female was found in the branchial cavity, specifically attached to the base of the gill cavity, facing the head towards anterior, and the dorsal body closely adpressed against the gill, while the ventral brood presses against the inner wall of the operculum (see fig. 1). Males were found to occupy the opposite gill chamber in the same position.

***Brucethoa alvaradoensis***  **(Rocha-Ramírez, Chávez-López & Bruce, 2005) comb. nov.**

*Elthusa alvaradoensis*.—Rocha-Ramírez, Chávez-López & Bruce, 2005: 701–707, figs 1–2.

***Host:*** *Synodus foetens* (Linnaeus) (Rocha-Ramírez et al., 2005).

***Distribution:*** Central Veracruz, Mexico (Rocha-Ramírez et al., 2005).

**Remarks:** *Brucethoa alvaradoensis* (Rocha-Ramírez, Chávez-López & Bruce, 2005) comb. n. Initially described as *Elthusa alvaradoensis* by Rocha-Ramírez, Chávez-López & Bruce, 2005, based on the materials collected from the branchial cavity of inshore lizardfish, *Synodus foetens* (Linnaeus, 1766), was collected from the coast of central Veracruz, Mexico. Based on the following characters: antennula narrowly separated by rostrum, buccal cone obscuring antennal bases, pleopods rami all simple, without folds or thickened ridges, coxal plates not conspicuous in dorsal view, relatively wide pleon, with lateral gaps between pleonites makes it fit with the new genus *Brucethoa.*

***Brucethoa bharata*** **Aneesh, Hadfield, Smit & Kumar, 2020**

(Fig. 21)

**Fig. 21:**
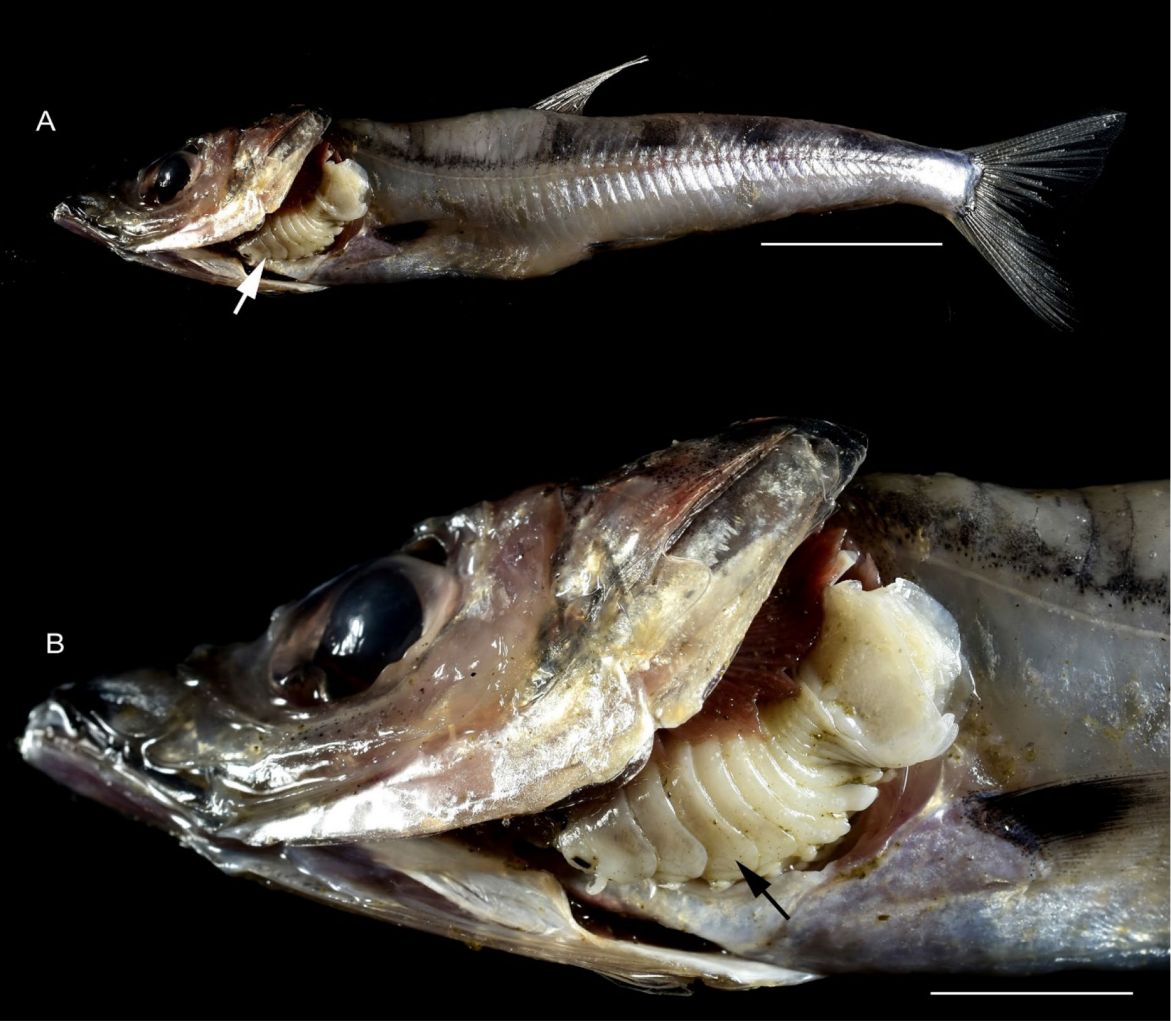
**A–B.** *Brucethoa bharata* Aneesh, Hadfield, Smit & Kumar, 2020 ovigerous female on the branchial cavity of the host fish *Glossanodon macrocephalus* Bineesh & Endo. *Scale, A: 20 mm; B: 10 mm.*

*Brucethoa bharata*.—Aneesh, Hadfield, Smit & Kumar, 2020: 565–584, figs 1–12.— Aneesh, Helna & Kumar, 2021d: 343, fig. 1c.

***Material examined:*** 17 ♀♀; 8 ♂♂ all from *Glossanodon macrocephalus* Bineesh and Endo, coll. PT Aneesh from Neendakara, Kollam, southwest coast of India.

***Voucher specimens:*** 1 ♀ (ovigerous) (20 mm TL) (Reg. No. CAH/INV/ISO 0306); 1♀ (ovigerous) (23 mm TL), (Reg. No. CAH/INV/ISO 0307); 1 ♂ (9 mm TL) (Reg. No. CAH/INV/ISO 0308).

***Host:*** *Glossanodon macrocephalus* Bineesh & Endo(Argentinidae) (Aneesh et al., 2020b).

***Distribution:*** Muttom, southwest coast, India (type locality) (Aneesh et al., 2020b); Neendakara, Kollam district, Kerala state, southwest coast of India (present study).

**Fig. 22:**
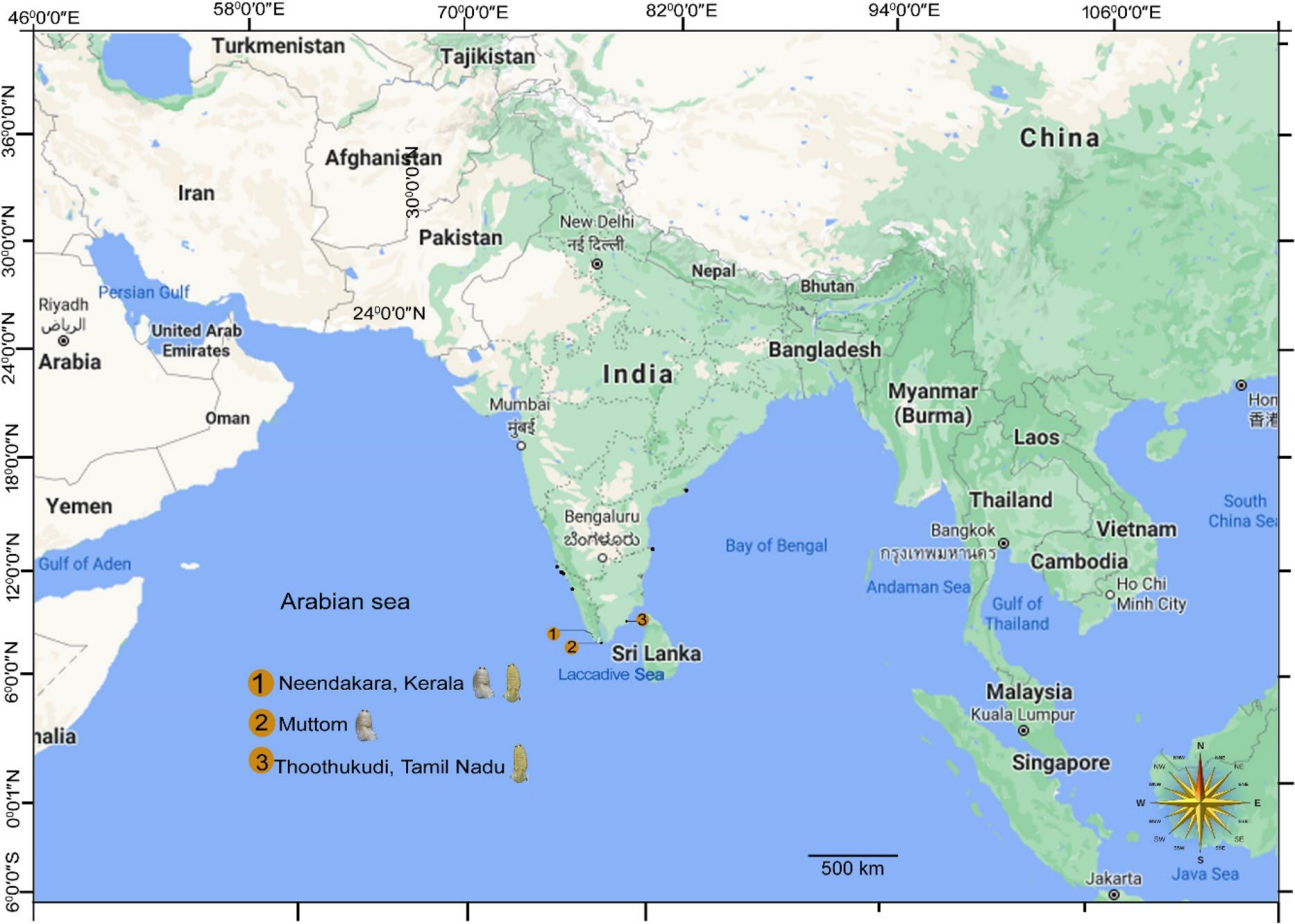
Map of the distribution of *Brucethoa bharata* Aneesh, Hadfield, Smit & Kumar, 2020 and *Brucethoa isro* **n. sp. (**1) Neendakara, Kerala coast; (2), Muttom, Tamil Nadu coast; (3), Thoothukudi, Tamil Nadu coast (source: https:// www.google.co.in/maps).

**Remarks: **Recently, Aneesh et al., (2020b) described *Brucethoa bharata,* from specimens collected from *Glossanodon macrocephalus* from the Muttom, southwest coast, India. In the present study, we have collected 17 females (eleven ovigerous and six non-ovigerous) and eight males of *Brucethoa bharata* from the same host fish, *G. macrocephalus,* from the new locality Neendakara, Kollam district, Kerala state. *Brucethoa bharata,* can be identified by the following combinations of characters: the strongly vaulted dorsum, pereonites 6 and 7 with posterolateral margin expanded and coxae not visible in dorsal view, relatively wide pleon that has gaps between the pleonites, large pleopod rami that extend laterally well beyond the pleotelson margins, ovigerous females have proximally thickened oostegites, and the marsupium is posteriorly partly enclosed by two sub-rectangular fleshy lobes (see Aneesh et al., 2020b) (Fig. 22).

***Brucethoa epinepheli (***Trilles & Justine, 2010**) comb nov.**

*Elthusa epinepheli*.—Trilles & Justine, 2010: 177–187, figs, 1–7.

***Host:*** *Epinephelus howlandi* (Günther) (Argentinidae) (Trilles & Justine, 2010).

***Distribution:*** New Caledonia, the Southwestern Pacific (Trilles & Justine, 2010).

**Remarks:** *Brucethoa epinepheli*  (Trilles & Justine, 2010) **comb. n.** was described initially based on the materials collected from the black-saddle grouper *Epinephelus howlandi* (Serranidae, Epinephelinae) from the coral reef of New Caledonia, the Southwestern Pacific by Trilles & Justine (2010), in the genus *Elthusa.* Based on the following characters: cephalon immersed in pereonite 1, coxae shorter than or as long as pereonites, relatively wide pleon, antennula narrowly separated by rostrum, buccal cone obscuring antennal bases, pleopods rami all simple, without folds or thickened ridges, sternite 7 with prominent posterior lobes, we place the species in combination with *Brucethoa.*

**Key to the global species of** ***Brucethoa*** **Aneesh, Hadfield, Smit & Kumar, 2020**

1. Body 2–2.1 times as long as wide; uropod exopod shorter than endopod………………………**2**
  Body more than 2.6 times as long as wide; uropod exopod as long as or longer than endopod………...**3**
2. Cephalon deeply immersed in pereonite 1; body widest at pereonite 3……………*** B. epinepheli***
  Cephalon not deeply immersed in pereonite 1; body widest at pereonite 6…………***B. bharata***
3. Pleotelson 1.3 times as wide as long; antenna with 10 articles; cephalon weakly immersed in pereonite 1……………………………………***B. isro*** **sp. nov.**
  Pleotelson 1.5 times as wide as long; antenna with 12–14 articles; cephalon not immersed in pereonite 1…………………………………***B. alvaradoensis***

## Data Availability

Type and voucher specimens were deposited in the collections of Western Ghat Field Research Centre of Zoological Survey of India, Kozhikode (ZSI/WGRC).
